# A fungal endophyte induces transcription of genes encoding a redundant fungicide pathway in its host plant

**DOI:** 10.1186/1471-2229-13-93

**Published:** 2013-06-26

**Authors:** Sameh SM Soliman, Christopher P Trobacher, Rong Tsao, John S Greenwood, Manish N Raizada

**Affiliations:** 1 Department of Plant Agriculture, University of Guelph, Guelph, ON N1G 2W1, Canada; 2 Faculty of Pharmacy, Zagazig University, Zagazig, Egypt; 3 Department of Molecular and Cellular Biology, University of Guelph, Guelph, ON N1G 2W1, Canada; 4 The Guelph Food Research Centre, Agriculture and Agri-Food Canada, Guelph, ON N1G 5C9, Canada

**Keywords:** Taxus, Paraconiothyrium, Fungus, Endophyte, Taxol, Biosynthesis, Fungicide, DXR, Taxadiene synthase

## Abstract

**Background:**

Taxol is an anti-cancer drug harvested from *Taxus* trees, proposed ecologically to act as a fungicide. *Taxus* is host to fungal endophytes, defined as organisms that inhabit plants without causing disease. The *Taxus* endophytes have been shown to synthesize Taxol *in vitro*, providing *Taxus* with a second potential biosynthetic route for this protective metabolite. Taxol levels in plants vary 125-fold between individual trees, but the underlying reason has remained unknown.

**Results:**

Comparing *Taxus* trees or branches within a tree, correlations were observed between Taxol content, and quantity of its resident Taxol-producing endophyte, *Paraconiothyrium* SSM001. Depletion of fungal endophyte *in planta* by fungicide reduced plant Taxol accumulation. Fungicide treatment of intact plants caused concomitant decreases in transcript and/or protein levels corresponding to two critical genes required for plant Taxol biosynthesis. Taxol showed fungicidal activity against fungal pathogens of conifer wood, the natural habitat of the Taxol-producing endophyte. Consistent with other Taxol-producing endophytes, SSM001 was resistant to Taxol.

**Conclusions:**

These results suggest that the variation in Taxol content between intact *Taxus* plants and/or tissues is at least in part caused by varying degrees of transcriptional elicitation of plant Taxol biosynthetic genes by its Taxol-producing endophyte. As Taxol is a fungicide, and the endophyte is resistant to Taxol, we discuss how this endophyte strategy may be to prevent colonization by its fungal competitors but at minimal metabolic cost to itself.

## Background

Endophytes are typically fungi and bacteria that inhabit plants without causing disease symptoms [1]. Some endophytes have been shown to benefit their hosts by improving nutrient availability, overcoming abiotic stress and as biocontrol agents against plant pathogens [2,3]. In *Lolium* grasses, the fungal endophyte, *Neotyphodium coenophialum*, has been shown to protect its host against herbivory by production of a loline alkaloid, creating a symbiotic relationship [4]. Taxol is a diterpenoid anti-cancer drug harvested from *Taxus* (yew) trees [5,6]. Ecologically, Taxol is proposed to act as a fungicide [7]. Interestingly, *Taxus* plants have been shown to host Taxol-producing fungal endophytes [8]. Taxol is reported to be synthesized by at least 18 different fungal genera, primarily endophytes that live symptomlessly within *Taxus* and other trees [8,9]. Fungal Taxol has been identified using HPLC/MS, a plant Taxol monoclonal antibody and NMR [10-12]. Though controversy exists whether plant compounds are required for its synthesis [13], fungal Taxol is produced independently of the plant following several rounds of *in vitro* culturing [10,14,15]. Recently we isolated a Taxol-producing fungal endophyte strain, *Paraconiothyrium* SSM001 from *Taxus x media*, and similarly demonstrated that it could produce Taxol *in vitro* independently of its host [16,17].

Plant Taxol consists of a taxane ring derived from the methylerythritol phosphate (MEP) pathway with a phenylalanine-derived side chain [18]. Other reports have suggested that the mevalonate pathway contributes to Taxol biosynthesis in older plants [19,20]. HMGR and DXR are rate-limiting enzymes in the MEP and mevalonate pathways, respectively [21]. We have recently demonstrated that fungi may similarly utilize both mevalonate and non-mevalonate pathways for Taxol biosynthesis [16], suggestive of redundancy in the plant and fungal biosynthetic pathways. Plant Taxol biosynthesis has been shown to require an additional ≥19 biosynthetic steps, with the committed step being taxadiene synthase (TS; EC 4.2.3.17) [18].

It has been known that Taxol levels in intact trees vary by up to 125-fold from tree to tree [22-26], but the underlying reason has never been addressed. Specifically, plant Taxol levels vary by species [24] and even from tree to tree within a species; amongst other factors, the latter variation has been associated with tree age [22,23] or season of sampling [27,28]. As one possible mechanism, Taxol-producing fungal endophytes have been shown to elicit Taxol production *in vitro* from *Taxus* plant suspension cultures [29]. Co-culturing of *Taxus chinensis* with *Fusarium mairei* fungus caused a 38-fold increase in a co-culture system [29], while *F. mairei* fungal broth caused a two-fold increase in Taxol production from *Taxus cuspidata* suspension cultures [29]. In other studies, fungi have also been shown to elicit the production of other terpenoids *in planta*[30-32]. The mechanism of Taxol elicitation is not known, and there is no evidence that these fungi also elicit plant Taxol production in intact plants.

Based on the previous literature, here we tested the ecological hypothesis that the dramatic variation in plant Taxol content may be caused by underlying variation in its Taxol-producing fungal endophytes acting as elicitors of enzymes in the plant Taxol biosynthetic pathway.

## Results

### Tree to tree variation in plant Taxol concentration correlates with the quantity of its resident fungal endophytes

**Figure 1:**
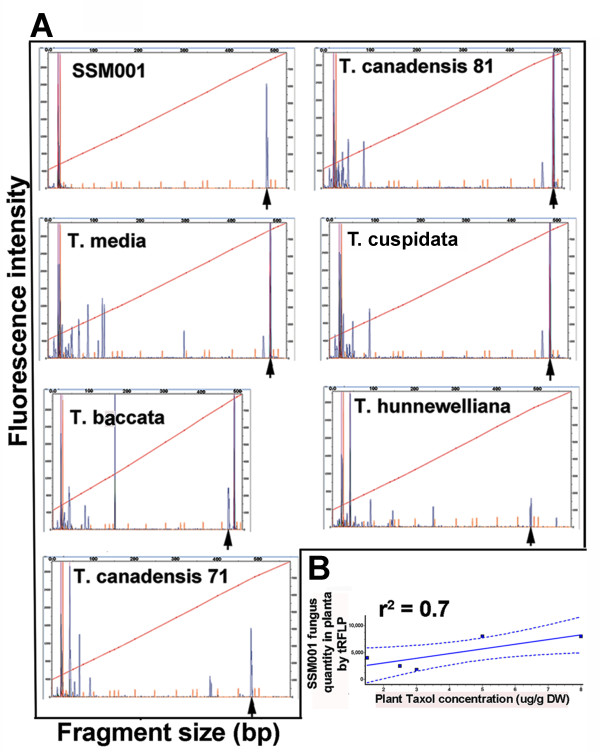
**Tree to tree variation in plant Taxol concentration correlates with the quantity of its resident *Paraconiothyrium *SSM001 fungal endophyte.** For ecological sampling, single ~5-6 cm stem pieces from a diversity of *Taxus* trees were harvested and cut into small pieces. The majority of the subsamples were pooled for DNA extraction or Taxol quantification, and the remainder were used for isolation of endophytic fungus. **(A)** tRFLP DNA fingerprinting of the fungal endophyte community in the stem sample of each *Taxus* species were used for fungal 18S rDNA primer-based amplification and restriction. Cultured Taxol-producing fungus, *Paraconiothyrium* SSM001, was used as the positive control. Nucleotide sizes on the x-axis identify unique fungal isolates. The y-axis indicates the quantity of the fungal isolate. **(B)** Corresponding correlation between plant Taxol yield from the wood samples taken from different *Taxus* species and the *Paraconiothyrium* endophyte quantity *in planta.* Identification and quantification of *Paraconiothyrium* endophyte were performed using tRFLP, a PCR-based technique which amplifies the entire fungal community using DNA collected from pooled plant tissue; amplicons are restriction digested, and the fragment size (x-axis, panel a) indicates the fungal strain; simultaneously, the size of the peak (y-axis, panel a) indicates the amount of fungus. For all experiments, a single 5–10 cm stem piece was harvested from each tree, which was divided into small sections of which ~8-10 were pooled for Taxol quantification and/or fungal DNA fingerprinting.

An initial result *in vitro* using a *T. x media* cell culture line showed a moderate correlation between plant Taxol accumulation *in vitro* and the concentration of its resident fungal endophytic community [see Additional file 1]. To determine if there was a correlation between Taxol content and Taxol-producing fungal endophyte(s) in intact plants, tissue samples were taken from six nearby *Taxus* trees, representing five plant species [*T. x hunnewelliana, T. canadensis, T. x media, T. cuspidata* (S. et Z.), and *T. baccata* (L.)]. Wood samples from each species were found to contain an endophytic fungus. Each fungus was cultured to purity; 18S rDNA sequencing showed that the identity of all fungi corresponded to *Paraconiothyrium*, with ~1% nucleotide substitutions in comparison to SSM001 ITS sequence [see Additional file 2], a Taxol-producing fungal strain that we recently characterized from local *T. x media* plants [16]. As different endophytes could be present in each plant, the level of SSM001 fungus in each stem sample was measured semi-quantitatively using tRFLP-based fungal community 18S rDNA fluorescence fingerprinting. TRFLP is a PCR-based method which amplifies the entire fungal community using DNA pooled from plant tissue; amplicons are restriction digested, and the fragment size (x-axis, Figure 1A) indicates the fungal genotype, while the size of the peak (y-axis, Figure 1A) indicates the amount of fungus. A prior small-scale experiment showed that *in planta* fungal quantification using TRFLP was consistent with results using quantitative PCR [see Additional file 3]. Using tRFLP fingerprinting, cultured SSM001 showed a distinctive peak (490 nucleotide fragment) (Figure 1A, SSM001 panel) and the same peak was identified in all trees. The tRFLP peak sizes were used for quantification of SSM001 quantity *in planta* (Figure 1A, B). Plant Taxol levels from each pooled wood sample showed good correlation (Pearson coefficient r^2^= 0.70, P=0.0382) with the quantity of resident SSM001 (Figure 1B) [see Additional file 4].

### Variation in Taxol yield within a single *Taxus* tree branch correlates with micro-scale variation in the quantity of its resident Taxol-producing endophyte

**Figure 2:**
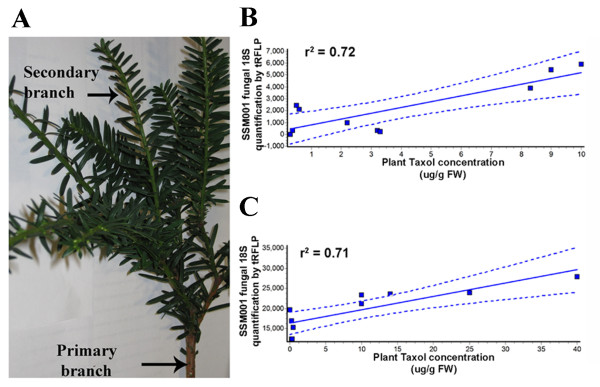
**Micro-scale plant tissue variation in Taxol quantity correlates with the quantity of resident Taxol-producing fungus. (A)** Picture of a *Taxus x media* primary branch with secondary branches that were sampled. Primary branches are the first branches which initiate from the main stem, whereas secondary branches initiate from primary branches. **(B, C)** Pearson correlation between fungal endophyte SSM001 quantity and plant Taxol concentration in **(B)** different adjacent primary branches of the same *Taxus* plant, and **(C)** adjacent secondary branches belonging to the same primary branch. Each primary or secondary branch was used for fungal 18S rDNA tRFLP fingerprinting or plant Taxol quantification using the Taxol immunoassay; each Taxol data point is the mean of 3 technical replicates of the immunoassay.

Since endophytic fungal populations within a tree may vary at a micro-scale, Taxol was measured in different adjacent primary branches within an individual *Taxus* plant (Figure 2A) and compared to the quantity of endophytic Taxol-producing fungus *Paraconiothyrium* SSM001 (measured by tRFLP semi-quantitative PCR). Plant Taxol accumulation in adjacent primary branches showed good correlation (Pearson correlation showed, r^2^=0.72, P=0.0037) with the quantity of SSM001 fungi (Figure 2B). The experiment was repeated at a finer scale by measuring Taxol in adjacent secondary branches belonging to the same primary branch (Figure 2A). Plant Taxol in adjacent secondary branches again correlated with the quantity of SSM001 fungi (Pearson correlation, r^2^=0.71, P=0.0021) (Figure 2C). These data suggest that fine scale variation in Taxol yield within intact *Taxus* trees correlates with local variation in the amount of Taxol-producing endophytic fungi.

### Endogenous fungi affect plant Taxol yield in both young and mature *Taxus* plants

**Figure 3:**
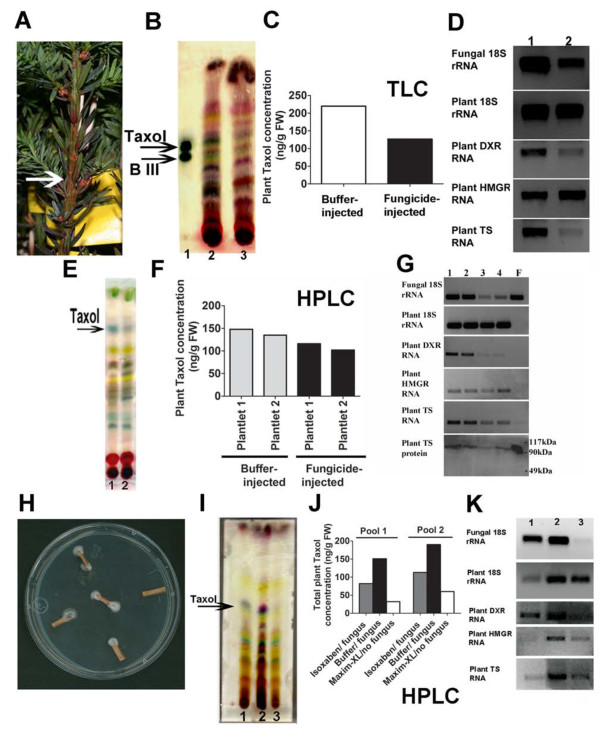
**Endogenous fungi contribute to plant Taxol yield in (A-G) young plantlets and (H-K) mature *Taxus *wood by eliciting plant Taxol pathway gene expression. (A-D)** Effect of injecting *T.x media* plantlets with Maxim fungicide. **(A)** Fungicide-injected plantlets showed systemic fungicide spread (red colour) from the injection site (arrow). (**B**) Taxane spectrum TLC for: Lane 1, standard Taxol and baccatin III (**B** III); Lane 2, buffer-injected plantlet; Lane 3, fungicide-injected plantlet. **(C)** Corresponding TLC quantification of plant Taxol. **(D)** Corresponding effects on endophytic fungal activity and expression of rate-limiting enzymes in the plant terpenoid and Taxol pathways: Lane 1, buffer-injected plantlet; Lane 2, fungicide-injected plantlet. **(E-G)** Effect of injecting two additional *T.x media* plantlets with fungicide and HPLC confirmation of Taxol. **(E)** Taxane spectrum TLC for: Lane 1, buffer injected plantlets, and Lane 2, fungicide-injected plantlet. **(F)** Corresponding HPLC quantification of plant Taxol. **(G)** Corresponding effects on endophytic fungal activity and expression of rate-limiting enzymes in the plant terpenoid and Taxol pathways: Lanes 1 and 2, two buffer-injected plantlets; Lanes 3 and 4, two fungicide-injected plantlets; Lane **F**, pure *Paraconiothyrium* SSM001 mycelia. **(H-K)** Effect of fungicide or herbicide on pools of *T.x media* mature cultured wood. For each of two biological replicates, 4–6 pieces were treated, pooled, and the data normalized by mass. **(H)** Cultured *T.x media* wood showing endophytic fungal growth. **(I)** TLC for cultured wood previously treated for 24 h with either herbicide (Lane 1, isoxaben, cellulose inhibitor), buffer (Lane 2) or fungicide (Lane 3, Maxim). **(J)** Corresponding Taxol HPLC quantification. **(K)** Corresponding effects on expression of endophytic fungal and plant 18S rRNA, and rate-limiting enzymes in the plant Taxol pathway (plant TS, taxadiene synthase) and plant terpenoid pathways (plant DXR, non-mevalonate pathway; plant HMGR, mevalonate pathway) (unit expression/plant fresh weight) [See Additional file 4 for statistics].

To characterize the effects of resident Taxol-producing fungi on the plant Taxol biosynthetic pathway in intact *Taxus* plants, we decided to treat plants with a potent fungicide, Maxim XL. The efficacy of Maxim XL fungicide on *Paraconiothyrium* SSM001 was verified *in vitro*: 0.1 μM was sufficient to completely inhibit the growth of *Paraconiothyrium* SSM001 on agar [see Additional file 5]. One-year-old *Taxus x media* plantlets were injected with different concentrations of fungicide (Maxim XL), and the injections were repeated over a three-month period (Figure 3A). Five months after the start of the experiment, the highest concentration of fungicide reduced plant Taxol accumulation (Figure 3B, C). The specificity of the fungicide was confirmed by decreased expression of fungal 18S rRNA in comparison to *Taxus* plant 18S rRNA which showed no change following fungicide treatment (Figure 3D). Additional plantlets were injected with the high fungicide concentration and again showed declines in the Taxol concentration based on TLC and HPLC measurements (Figure 3E, F).

To confirm the above results independently, the same fungicide (Maxim XL) was also applied to mature *Taxus* wood. Since it was not feasible to treat mature trees with fungicide, fresh wood pieces (4–6 per biological replicate pool) from a mature *T. x media* tree (Figure 3H, >30 years old) were instead harvested and treated *in vitro.* Wood pieces were exposed to fungicide or buffer for 24 h, incubated for two weeks and then pooled prior to analysis. The fungicide treatment again caused declines in plant Taxol based on TLC analysis (Figure 3I, Lane 3) and confirmed by HPLC (Figure 3J). The high specificity of the fungicide was again confirmed by decreased expression of fungal 18S rRNA compared to plant 18S rRNA (Figure 3K, Lane 3). However, the effect of the fungicide may have been artificially exaggerated in the mature wood experiment (75% decline in Taxol, Figure 3J) compared to the plantlet experiment (20% decline, Figure 3F) as the two-week *in vitro* wood incubation allowed growth of endophytic fungus in the buffer control (Figure 3H).

Two-way ANOVA (tissue type versus treatment) statistically confirmed that the fungicide treatments caused significant declines in plant Taxol [see Additional file 4]. These data suggested a significant relationship between resident endophytic fungi and plant Taxol production in nature.

### Endophytic fungi affect plant Taxol yield by eliciting transcription of plant Taxol biosynthesis genes

To determine whether the contribution by endophytic fungi to plant Taxol was the result of direct fungal biosynthesis of Taxol or elicitation of plant Taxol biosynthetic enzymes, expression of key genes in the plant Taxol pathway were assayed following the fungicide treatments in both young plantlets and mature wood. The diterpenoid taxane backbone of plant Taxol is derived from DXR, a key enzyme in the MEP pathway [33]. Fungicide treatment resulted in decreased levels of DXR transcript in both intact plantlets (Figure 3D, Lane 2 and Figure 3G, Lanes 3 and 4) and mature wood (Figure 3K, Lane 3). The expression of another plant enzyme was tested, HMGR, the rate-limiting step in the cytosolic mevalonate (MVA) pathway which contributes to the taxane ring but only in old *Taxus* trees [19,20]. Fungicide treatments caused no obvious decline in the expression of HMGR in young plantlets (Figure 3D, G), but caused a decline in older wood (Figure 3K**,** Lane 3). Finally, in both young plantlets and old *Taxus* wood, fungicide treatments caused a reduction in the expression of plant taxadiene synthase (TS), the rate-limiting step in the plant Taxol pathway [18] (Figure 3D, G and Figure 3K, Lane 3). This result was verified at the protein level using a polyclonal antibody for *Taxus* TS (Figure 3G). It was theoretically possible that some of the observed decline in TS protein may have been caused by decreased fungal TS as it cross-reacts with the plant antibody [see Additional file 6]. However, as the plant TS RT-PCR primers used never recognized pure cultures of *Paraconiothyrium* SSM001 TS (Figure 3G, Lane F), the simplest explanation is that endophytic fungi elicit plant TS transcription or transcript accumulation. It should also be noted that primers for plant HMGR and DXR did not amplify fungal endophyte RNA (Figure 3G, Lane F). Six months later, an independent set of *Taxus* plantlets was injected with Maxim-XL fungicide or buffer and showed similar results as the first set of experiments [see Additional file 7]. The primers used to quantify expression of *Taxus* taxadiene synthase (TS), DXR and HMGR, never amplified any fungal bands using fungal templates. Several other *Taxus* plant primers (regular or degenerate) designed from conserved sequences sometimes amplified a band, but further sequencing demonstrated that these fungal amplicons were not authentic TS, DXR or HMGR [see Additional file 8].

These independent results in young plantlets and old *Taxus* wood demonstrate that endophytic fungi contribute to plant Taxol yield by eliciting transcription of rate-limiting early and late steps in the plant Taxol pathway. However, these results do not exclude a direct biosynthetic contribution by fungi to plant Taxol.

### Application of herbicide suggests that the plant-endophyte interaction is critical for high plant Taxol accumulation

In parallel with the fungicide treatments, fresh *Taxus x media* mature wood pieces (Figure 3H) were simultaneously treated with isoxaben, a herbicide predicted to be specific for plants rather than fungi as it is a cellulose inhibitor [34]. Wood pieces were exposed to herbicide or buffer for 24 h, incubated for two weeks and then pooled (4–6 pieces per biological replicate) prior to analysis. Though the herbicide treatment caused a much greater decline in the expression of plant 18S rRNA, HMGR and TS compared to the parallel fungicide treatment (Figure 3K), plant Taxol yield was more affected by the fungicide treatment (Figure 3I, J) which reduced endophytic fungal 18S expression to a much greater extent (Figure 3K). Therefore, in this *in vitro* system, fungi made a greater contribution to Taxol yield than cultured wood alone. The highest Taxol yield occurred when both plant 18S rRNA and fungal 18S rRNA expression were high suggesting that the ecological interaction may be important for maximum Taxol biosynthesis *in planta*.

### Taxol inhibits growth of other fungal inhabitants of wood but not the Taxol-producing endophyte

**Figure 4:**
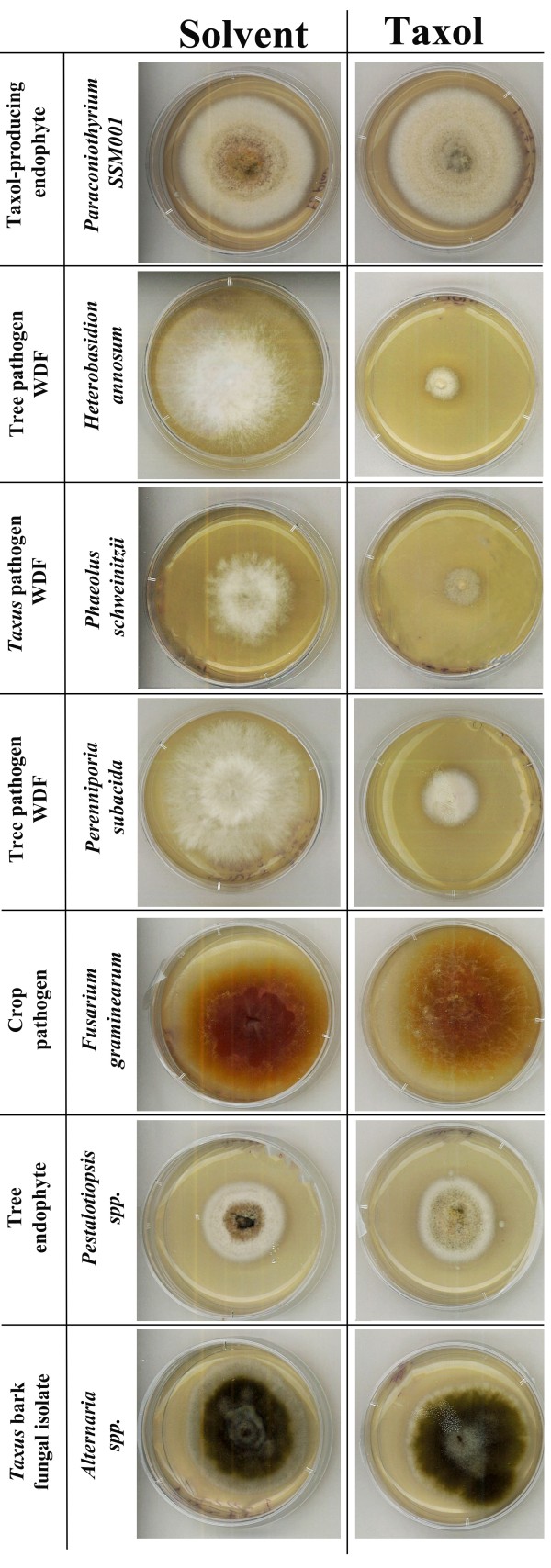
**The effect of Taxol on the growth of *Paraconiothyrium *SSM001, other endophytes and pathogens including wood decaying fungi.** Taxol was added to each fungus at a concentration of 12 μM, compared to the solvent control. Shown are the following fungi: *Taxus* endophyte *Paraconiothyrium* SSM001; three wood decaying fungi (*Heterobasidion annosum, Perenniporia subacida*, and the *Taxus* pathogen, *Phaeolus schweinitzii*); a crop pathogen (*Fusarium graminearum*)*,* a tree endophytic fungus (*Pestalotiopsis* spp.), and an isolate of *Taxus* bark (*Alternaria* ssp). Taxol only inhibited the growth of the three wood decaying fungi.

It was surprising that the fungal endophyte SSM001 would induce plant Taxol biosynthesis *in planta*, given that the endophyte can produce the same metabolite by itself, at least *in vitro*[16]. As Taxol was reported to have fungicidal activity, we hypothesized that SSM001 might stimulate plant Taxol in order to inhibit its fungal competitors *in planta*. Strain SSM001 was isolated from *Taxus* wood. We applied Taxol (HPLC grade, Sigma #T7402 isolated from *Taxus brevifolia*) to three different fungi known to infect conifer wood: *Heterobasidion annosum*, *Phaeolus schweinitzii* and *Perenniporia subacida*, of which *P. schweinitzii* is known to infect *Taxus* wood [35-37]. Taxol inhibited the growth of all three wood decaying fungi (Figure 4). However, Taxol did not inhibit growth of an *Alternaria* fungus that we had isolated earlier from *Taxus* bark, nor a tree endophyte (*Pestalotiopsis* ssp.) or a non-tree pathogen of corn, *Fusarium graminearum* (Figure 4). Critically, Taxol also did not inhibit growth of the SSM001 endophyte (Figure 4). This data suggests that Taxol acts as a fungicide against potential fungal competitors of endophyte SSM001 that might share its wood environment, though not against the endophyte itself.

## Discussion

Several earlier reports showed that Taxol levels in suspension cultures [38] and intact trees [23,24] vary by up to 125-fold, but the underlying reason has never been shown. Independent studies showed that Taxol-producing fungal endophytes elicit the production of Taxol from *in vitro Taxus* cell cultures [29], though did not determine the mechanism of elicitation. Here we present data that these two phenomena are mechanistically related. Specifically, we have shown that tree-to-tree, and even branch-to-branch, variation in *Taxus* plant Taxol accumulation positively correlates with the quantity of a resident Taxol producing fungal endophyte, in this case *Paraconiothyrium* SSM001 (Figure 1, 2). The underlying mechanism involves endophyte-induced transcriptional activation of rate limiting genes in the plant Taxol biosynthetic pathway (Figure 3).

Given that the natural environment of the endophytic fungal strain is *Taxus* wood, and that Taxol was reported to be a fungicide [39], we hypothesized that the endophyte might stimulate its host to produce Taxol in order to inhibit fungal competitors that could also colonize the same woody environment. Consistent with this hypothesis, Taxol inhibited growth of fungi known to infect conifer wood including *Taxus* wood (Figure 4).

Nevertheless, this raises an important question as to why the endophyte needs to stimulate the host plant to produce Taxol when the endophtye can produce the same secondary metabolite by itself, at least *in vitro*[16]. We propose that this endophyte strategy may be to prevent colonization by its competitors but at minimal metabolic cost to itself. We have recently shown that Taxol biosynthesis in this fungal endophyte (*Paraconiothyrium* SSM001) consumes expensive terpenoid and phenylpropanoid pathway metabolites [16]. Such a relationship between *Taxus* and its fungal endophyte, wherein the endophyte takes advantage of its host, would represent an interesting example of commensalism.

These results raise the reverse question: why does the *Taxus* host retain an endophyte that produces a secondary metabolite that it can synthesize on its own? Our results even demonstrate that an antibody raised to recognize taxadiene synthase (TS), the rate limiting step in plant Taxol biosynthesis, strongly cross reacts to the apparent endophyte TS (Figure 3G), a result which appeared to be confirmed using a negative control (non-Taxol producing *Fusarium* fungus) and positive controls (two fungal Taxol elicitors) (Additional file 6). This result suggests a shared evolutionary relationship between plant and fungal endophyte Taxol biosynthetic pathways, rather than convergent evolution. One attractive possibility is that if a pathogen were able to successfully colonize the host, the plant may not be able to produce Taxol locally due to the resulting plant tissue damage. By the host maintaining a Taxol-producing endophyte, even at a metabolic cost to itself in the short-term, it may afford itself long-term protection against systemic infection in situations where it can no longer produce the Taxol fungicide – a temporal form of symbiosis. Testing this hypothesis would require being able to chemically trace the biosynthetic origin of Taxol *in planta* before and after a fungal pathogen infection.

Other fungi have also been shown to elicit plants to produce terpenoid-based compounds *in planta*[30-32]. Arbuscular mycorrhizal fungi induce the accumulation of mycorradicin in gramineous plant roots by inducing 1-deoxy-D-xylulose 5-phosphate synthase (DXS) and DXR, two key enzymes in the MEP pathway [30,31]. Terpenoid production in *Euphorbia pekinensis* (Rupr) plants increases upon inoculation with the endophytic fungus, *Phomopsis* sp., by induction of plant phenylalanine ammonia-lyase (PAL) and DXR. Finally, fungal elicitors have been shown to induce biosynthesis of the anti-malarial terpenoid, artemisinin, in *Artemisia annua* (L.) plant suspension cells [32].

On a cautionary note, as only a modest correlation was observed between SSM001 and plant Taxol concentrations, and since the fungicide treatments did not specifically target SSM001, it is possible that other fungi within *Taxus* might also elicit plant Taxol biosynthesis, consistent with Taxol acting as a fungicide. Recently, we also demonstrated that other fungi inhabiting *Taxus* can elicit Taxol production from the endophytic fungus *in vitro*[17], raising the possibility of complex elicitor interactions on Taxol accumulation *in planta*.

## Conclusions

Taxol levels in intact *Taxus* trees were known to vary considerably from tree to tree but for unknown reasons. Comparing five *Taxus* species, good correlation was observed between plant Taxol content and the quantity of its Taxol-producing fungal endophyte. Measuring different adjacent branches within an individual *Taxus* plant, Taxol yield correlated with micro-scale variation in the amount of the endophyte. Fungicide treatment of intact *Taxus* plants or wood caused declines in both endophyte concentrations as well as plant Taxol accumulation, consistent with the correlation results. The fungal endophyte was found to affect plant Taxol yield by eliciting transcription of rate-limiting genes in the plant Taxol biosynthetic pathway. Taxol was an effective fungicide against fungal pathogens of conifer wood, the natural habitat of the Taxol-producing endophyte. The endophyte itself was resistant to Taxol. These results show that the fungal endophyte stimulates its host to produce a fungicide to which it is resistant, perhaps to prevent colonization by its competitors but at minimal metabolic cost to itself. To the best of our knowledge, the Taxol-producing fungal endophyte-*Taxus* relationship represents an unusual type of plant-endophyte interaction reported in nature, wherein both partners synthesize and potentially benefit from the same secondary metabolite.

## Methods

The general experimental design, reagents and materials are described in an Additional file [see Additional file 9].

### Isolation of endophytic fungi and fungal genotyping

Previously, a Taxol-producing endophytic fungus was cultured from old branches of *Taxus x media* plants cultivated on the University of Guelph Main Campus and Arboretum [16] (Guelph, Canada). The fungal ITS sequence matched to *Paraconiothyrium* spp [see Additional file 2], and the fungus was assigned the name *Paraconiothyrium* SSM001 [16]. For taxonomic classification and to ensure consistency between experiments, every fungal culture was genotyped by PCR and DNA sequencing of the internal transcribed spacer regions (ITS) of 18S rDNA [40] to confirm both strain identity and purity [see Additional file 9 for details].

### Terminal restriction fragment length polymorphism (tRFLP)

Specific fluorescent labelled fungal 18S primers were used for amplification. The primer sequences were nu-SSU-0817 5`-TTAGCATGGAATAATRRAATAGGA-3’ and nu-SSU-1536 5’-ATTGCAATGCYCTATCCCCA-3’ which amplify a 762 bp fragment [41]. TRFLP separation analysis used a capillary electrophoresis ABI prism 310 DNA Sequencer (PE Applied Biosystems, Canada). TRFLP data was analyzed using ABI Prism 310 Collection version 2.0 and Peakscanner Analysis Software (version 1.0) [see Additional file 9 for details].

### Taxol quantification

Taxol was verified and quantified either by a competitive immunoassay procedure [42,43], TLC spot densitometry [44,45] or by HPLC-UV. Additional details have been published [16] and are in an Additional file [see Additional file 9].

### Maxim XL fungicide effect on *Paraconiothyrium* SSM001

PDA plates were mixed with different concentration of the Maxim XL fungicide (0, 0.1, 0.5, 1.0, 1.5, 2.0, 2.5, 5.0 μM), and left to dry. A small piece of fungal mycelia was cultured separately on each plate surface and the plates were kept in the dark at room temperature for two weeks. Fungal mycelial growth was observed by the naked eye.

### Effect of fungicide on *Taxus x media* plantlets

In a pilot experiment, three different volumes (200 μL/plant, 500 μL/plant and 1 mL/plant) of Maxim XL fungicide (Syngenta) were injected into stems of one-year old *Taxus x media* plantlets or buffer (water). This was followed by RNA extraction and quantification of fungal endophytes by RT-PCR using specific fungal primers. In comparison to buffer injected plantlets, only 1 mL of fungicide/ plant was effective in decreasing the amount of endophytic fungi including *Paraconiothyrium* SSM001 using fungal 18S quantification. Subsequently, one-year old *T. x media* plantlets were injected with 1 mL/plant fungicide into the stem just passing beyond the outer bark. The plantlets received another two doses of the fungicide two months apart. The plantlets were left to grow for another 5 months with weekly watering. The plantlets were kept at room temperature (25°C) under 24 h cool white fluorescent light (60–80 μmol m^-2^ sec^-2^). Both the control and treated plantlets were arranged randomly. Only the stems were analyzed.

### Effects of fungicide and herbicides on Taxol yield

For each biological replicate, 4–6 wood pieces randomly pooled from fresh *T. x media* branches of mature trees obtained from the University of Guelph Campus, were incubated with 15 μM of either Maxim-XL fungicide (consists of 25 g/L fludioxonil, 10 g/L metalaxyl-M), isoxaben herbicide (N-(3-(1-ethyl-1-methylpropyl)-5-isoxazolyl)-2,6-dimethoxy benzamide powder), or buffer (water) for 24 h in the dark at 25°C with shaking at 50 rpm. The wood pieces were then washed with water three times and then sterilized as mentioned earlier prior to culturing on PDA medium for 2 weeks. Four grams of each treated wood were ground, and 50 mg was used for RNA extraction; the remainder was used for taxane extraction and Taxol quantification.

### Taxol fungicide assay

To test for the fungicide effects of Taxol, *Paraconiothyrium* SSM001 and six plant fungal pathogens and endophytes were incubated separately on PDA plates containing Taxol or the solvent control. Based on the literature and testing serial concentrations of Taxol (data not shown), 12 μM Taxol was chosen as the IC50. The following fungal pathogens were chosen: *Heterobasidion annosum*, *Perenniporia subacida,* an *Alternaria* ssp., a *Pestalotiopsis* ssp., *Phaeolus schweinitzii* and *Fusarium graminearum*. In all the experiments, the fungal plates were incubated in the dark at 25°C and the fungal growth was followed for one week.

### RT-PCR

For the gene, 3-hydroxy-3-methyl glutaryl CoA reductase, primers PlantHMGRF2 (5’-TCCCTGTGGGTGTTGCAGGGC-3’) and PlantHMGRR2 (5’-AACCTAACAACGGAGCCC -3’) were used. For 3-deoxy xylulose-5-phosphate reductoisomerase (DXR), primers PlantDXRF4 (5’-AGGTGGAACCATGACTGG-3’) and PlantDXRR4 (5’-TGCAGCATACTTTCTGGCCC-3’) were used. For taxadiene synthase, primers TSinF (5’-GGTTTGCTCCAAATCAGGGC-3’) and TSinR (5’-TAACATTGTGGTGCCACAGA-3’) were used. For fungal quantification, fungal 18S rRNA specific primers were used: 18SrDNA-RtF (5’-GGCATCAGTATTCAGTTGTC-3’) and 18SrDNA-RtR (5’-GTTAAGACTACGACGGTATC-3’) [46]. *Taxus* 18S rRNA was used as an internal standard for normalization using the following primers: Tax18SF2 (5’-TTTTCCCTTTGCAATGCC-3’) and Tax18SR2 (5’-TCGCCCTTGTAATAACCCG-3’) [see Additional file 9 for details].

### Identification of taxadiene synthase (TS) enzyme by Western blot analysis

Proteins were extracted from liquid nitrogen-ground *Taxus* stem tissues using extraction buffer [47] supplemented with 1% glycine [48] followed by SDS-PAGE on 12% acrylamide gels [49] and transferred to nitrocellulose membrane [50] followed by immunoblotting using a polyclonal anti-TS antibody [see Additional file 9 for details].

## Competing interests

The authors declare that they have no financial competing interests.

## Authors’ contributions

SSMS and MNR designed the research, analyzed the data and wrote the paper. SSMS performed the research. RC helped on the LC-MS and HPLC analysis. CPT and JG helped with the Western blot analysis. All authors read and approved the final manuscript.

## Supplementary Material

Additional file 1 **Variation in Taxol content in different *Taxus *suspension cultures moderately correlates with the quantity of its endophytic resident fungi.** (A-C) *Taxus x media* callus culture on B5CA medium at different stages. (A) 14 days post-culturing; (B) 18 days post-culturing; and at (C) 30 days post-culturing. (D) Pearson correlation between resident fungal quantity and plant Taxol concentration within 10 different suspension culture flasks created from the same *Taxus x media* plant cell culture line. Each tissue culture flask was sampled once for RNA and Taxol. Click here for file

Additional file 2 **ITS sequence of the Taxol-producing endophyte *Paraconiothyrium *SSM001.** Click here for file

Additional file 3 **Comparison between qRT-PCR and TRFLP methods to quantify the total fungal community within wood, bark and needles of *Taxus x media *plants.** Click here for file

Additional file 4 **Raw data used to statistically analyze the correlation between Taxol concentrations in stem samples taken from diverse *Taxus *species and *Paraconiothyrium *quantity *in planta.*** Click here for file

Additional file 5 **Effect of Maxim XL fungicide on *Paraconiothyrium *SSM001. 0.1 μM Maxim XL was sufficient to completely inhibit the growth of *Paraconiothyrium *SSM001.** Click here for file

Additional file 6 **Western blot detection of a protein from endophyte *Paraconiothyrium *SSM001 that cross reacts to a taxadiene synthase polyclonal antibody.** Lanes 1, 2: *Taxus* needle protein extract (positive control). Lane 3: *Fusarium* protein extract (negative control). Lane 4: Protein extract from SSM001 liquid culture co-cultured with wood decaying fungi (fungal Taxol elicitor). Lane 5: Protein extract from SSM001 culture treated with chloromethane (fungal Taxol elicitor). Lane 6: Protein extract from untreated SSM001 culture. Equivalent amounts of total protein were loaded onto each lane. Click here for file

Additional file 7 **Effect of injecting additional *Taxus x media *plantlets with fungicide versus buffer control. **Effects on fungicide on endophytic fungal activity and the expression of rate-limiting enzymes in the plant terpenoid and Taxol pathways: Lanes 1 and 2, two independent, buffer-injected plantlets; Lanes 3 and 4, two fungicide-injected plantlets; Lane F, pure *Paraconiothyrium* fungal mycelia. Shown are plant DXR (1-deoxy-D-xylulose-5-phosphate reducto-isomerase), the rate-limiting step in the non-mevalonate pathway; plant HMGR (3-hydroxy-3-methylglutaryl-coenzyme A reductase), rate-limiting step in the mevalonate pathway, and plant TS (taxadiene synthase), the rate-limiting step in the plant Taxol pathway [For statistical analysis see Additional file 4]. Click here for file

Additional file 8 **Sequences of amplified fungal bands using either fungal genomic DNA or RNA as templates in combination with PCR primers corresponding to *Taxus *plant taxadiene synthase, DXR and HMGR genes.** None of the amplified bands showed similarity to authentic taxadiene synthase, DXR or HMGR. Amplified bands using *Taxus* taxadiene synthase, DXR and HMGR primers sequences and *Taxus* DNA or cDNA templates were used as positive controls. Click here for file

Additional file 9 Additional methodology details, including: experimental design, error and replicates, isolation of endophytic fungi, fungal genotyping, terminal restriction fragment length polymorphism (tRFLP), Taxol quantification, callus and suspension culture initiation protocol, RT-PCR, quantitative real-time PCR, and identification of taxadiene synthase (TS) enzyme by Western blot analysis. Click here for file
