# Supplementary material for: A fungal endophyte induces transcription of genes encoding a redundant fungicide pathway in its host plant
Source: BMC Plant Biol. 2013 Jun 26;13:93. doi: 10.1186/1471-2229-13-93 (PMC3700885; doi:10.1186/1471-2229-13-93)
Supplement: Additional file 4 — Raw data used to statistically analyze the correlation between Taxol concentrations in stem samples taken from diverse Taxus species and Paraconiothyrium quantity in planta. [file 1471-2229-13-93-S4.doc]

**Additional file 4: Raw data used to statistically analyze the correlation between Taxol concentrations in stem samples taken from diverse *Taxus* species and *Paraconiothyrium* quantity *in planta***.

| **Expt Name** | **Figure** | **Replicate Unit** | **Replicate Details** | | | **Assay** | | | | **Units** | | | ***Control Details*** | | | **Taxol concentration** | | **Treatment Details** | **Treatment Data** | **Statistical Test, Values and Significance** | | | | | | | | |
| --- | --- | --- | --- | --- | --- | --- | --- | --- | --- | --- | --- | --- | --- | --- | --- | --- | --- | --- | --- | --- | --- | --- | --- | --- | --- | --- | --- | --- |
| 1. Correlation between Taxol concentration in plants vs fungal quantity | Fig. 1(b) | Pooled stem pieces of several branches | Different *Taxus* spp | | | Plant taxol measured by Taxol immunoassay and fungal quantity measured by tRFLP | | | | plant Taxol in µg/g and fungal quantity in fluorescent units | | | *T. x hunnewelliana* | | | 3.1 | | SSM001 tRFLP peak | 1800 | Linear Correlation (Pearson)  Number of points = 6  Correlation coefficient (r) = 0.84  95% confidence interval: 0.07544 to 0.9816  Coefficient of determination (r squared) = 0.70  Two-tailed P value = 0.0382 (significant) | | | | | | | | |
|  |  |  |  |  |  |  |  |  |  |  |  |  | *T. cuspidata* | | | 5.12 | | SSM001 tRFLP peak | 8000 |  |  |  |  |  |  |  |  |  |
|  |  |  |  |  |  |  |  |  |  |  |  |  | *T. canadensis 71* | | | 1.5 | | SSM001 tRFLP peak | 4020 |  |  |  |  |  |  |  |  |  |
|  |  |  |  |  |  |  |  |  |  |  |  |  | *T. canadensis 81* | | | 8.16 | | SSM001 tRFLP peak | 8000 |  |  |  |  |  |  |  |  |  |
|  |  |  |  |  |  |  |  |  |  |  |  |  | *T. baccata* | | | 2.49 | | SSM001 tRFLP peak | 2490 |  |  |  |  |  |  |  |  |  |
|  |  |  |  |  |  |  |  |  |  |  |  |  | *T. x media* | | | 8.2 | | SSM001 tRFLP peak | 8000 |  |  |  |  |  |  |  |  |  |
|  |  |  | | |  | |  | | | |  | |  | | |  | |  |  | |  | |  | | | |  | |
| 2. Correlation between plant Taxol and fungal quantity | Fig. 2(b) | 10 different pooled plant stem pieces of different primary branches | | Same *T. x media* plant | | Taxol immunoassay | | ng/g | | | | P1 | | | | 10.00 | | Fungal quantity measured by tRFLP in each branch sample | 5920 | | Linear Correlation (Pearson)  Number of points = 10  Correlation coefficient (r) = 0.85  95% confidence interval: 0.4612 to 0.9626  Coefficient of determination (r squared) = 0.72  Two-tailed P value = 0.0037 (very significant) | | | | | | | |
|  |  |  |  |  |  |  |  |  |  |  |  | P2 | | | | 2.20 | |  | 1000 | |  |  |  |  |  |  |  |  |
|  |  |  |  |  |  |  |  |  |  |  |  | P3 | | | | 0.60 | |  | 2090 | |  |  |  |  |  |  |  |  |
|  |  |  |  |  |  |  |  |  |  |  |  | P4 | | | | 3.30 | |  | 210 | |  |  |  |  |  |  |  |  |
|  |  |  |  |  |  |  |  |  |  |  |  | P5 | | | | 0.30 | |  | 20 | |  |  |  |  |  |  |  |  |
|  |  |  |  |  |  |  |  |  |  |  |  | P6 | | | | 0.37 | |  | 310 | |  |  |  |  |  |  |  |  |
|  |  |  |  |  |  |  |  |  |  |  |  | P7 | | | | 9.00 | |  | 5420 | |  |  |  |  |  |  |  |  |
|  |  |  |  |  |  |  |  |  |  |  |  | P8 | | | | 0.50 | |  | 2420 | |  |  |  |  |  |  |  |  |
|  |  |  |  |  |  |  |  |  |  |  |  | P9 | | | | 3.20 | |  | 310 | |  |  |  |  |  |  |  |  |
|  |  |  |  |  |  |  |  |  |  |  |  | P10 | | | | 8.30 | |  | 3905 | |  |  |  |  |  |  |  |  |
|  |  |  | |  | |  | |  | | | |  | | | |  | |  |  | |  | | | | | | | |
| 3. Correlation between plant Taxol and fungal quantity | Fig. 2(c) | 10 different pooled plant stem pieces of different secondary branches | | Same primary branch of *T. x media* | | Taxol immunoassay | | ng/g | | | | P11 | | | 0.5 | | Fungal quantity measured by tRFLP in each branch sample | | 15290 | | Linear Correlation (Pearson)  Number of points = 10  Correlation coefficient (r) = 0.84  95% confidence interval: 0.4571 to 0.9623  Coefficient of determination (r squared) = 0.71  Two-tailed P value = 0.0021 (very significant) | | | | | | | |
|  |  |  |  |  |  |  |  |  |  |  |  | P12 | | | 0.26 | |  |  | 12420 | |  |  |  |  |  |  |  |  |
|  |  |  |  |  |  |  |  |  |  |  |  | P13 | | | 40 | |  |  | 27860 | |  |  |  |  |  |  |  |  |
|  |  |  |  |  |  |  |  |  |  |  |  | P14 | | | 25 | |  |  | 23940 | |  |  |  |  |  |  |  |  |
|  |  |  |  |  |  |  |  |  |  |  |  | P15 | | | 14 | |  |  | 23690 | |  |  |  |  |  |  |  |  |
|  |  |  |  |  |  |  |  |  |  |  |  | P16 | | | 0.2 | |  |  | 16920 | |  |  |  |  |  |  |  |  |
|  |  |  |  |  |  |  |  |  |  |  |  | P17 | | | 10 | |  |  | 23380 | |  |  |  |  |  |  |  |  |
|  |  |  |  |  |  |  |  |  |  |  |  | P18 | | | 0.002 | |  |  | 19640 | |  |  |  |  |  |  |  |  |
|  |  |  |  |  |  |  |  |  |  |  |  | P19 | | | 0.31 | |  |  | 12420 | |  |  |  |  |  |  |  |  |
|  |  |  |  |  |  |  |  |  |  |  |  | P20 | | | 10 | |  |  | 21120 | |  |  |  |  |  |  |  |  |
|  |  |  | |  | |  | |  | | | |  | | |  | |  | |  | |  |  | | | |  | | |
| 4. Effect of fungicide treatment on plant Taxol | Fig. 3(f) | Pooled stem pieces of one year old plantlets (4g) | *T. media* spp same variety | | | HPLC | | ng/g | | | | Buffer-injected | | 148 | | | Fungicide-injected (Maxim XL) | | 116.1 | | Two-way ANOVA | | | | | | | |
|  |  |  |  |  |  |  |  |  |  |  |  |  |  | 135 | | |  |  | 102 | | \| Source of Variation \| Df \| Mean square \| F \| P \| \| --- \| --- \| --- \| --- \| --- \| \| Interaction \| 1 \| 2968 \| 4.5 \| 0.1006 \| \| Treatment \| 1 \| 10075 \| 15 \| 0.0173 \| \| **Tissues** \| **1** \| **181** \| **0.28** \| **0.6269** \| \| Residual \| 4 \| 657 \|  \|  \| | | | | | | | |
|  | Fig. 3(j) | Pooled wood pieces of several branches | Same *T. media* branches | | | HPLC | | ng/g | | | | Buffer-treated | | 151 | | | Fungicide treated (Maxim XL) | | 32 | |  |  |  |  |  |  |  |  |
|  |  |  |  |  |  |  |  |  |  |  |  |  |  | 190 | | |  |  | 90 | |  |  |  |  |  |  |  |  |
|  |  |  |  | | |  | |  | | | |  | |  | | |  | |  | |  | | |  | | | |  |
| 5. Effect of fungicide and herbicide treatment on plant Taxol | Fig. 3(j) | Pooled wood pieces of several branches    (Pooled 4-6 wood) | Same *T. media* branches | | | HPLC | | | ng/g | | | | Buffer-treated  Lane 2 | | | 151 | Fungicide treated (Maxim XL)  Lane 3 | | 32 | | One-way Analysis of Variance (ANOVA) (ratio) | | | | P = 0.0047 | | | |
|  |  |  |  |  |  |  |  |  |  |  |  |  |  |  |  | 190 |  |  | 90 | |  |  |  |  |  |  |  |  |
|  |  |  |  |  |  |  |  |  |  |  |  |  |  | | | | Isoxabene herbicide treated (Fungus)  lane 1 | | 82 | | Comparison P value  ================================== Buffer-treated vs Fungicide treated P<0.01  Buffer-treated vs isoxaben (fungus) P<0.05 | | | | | | | |
|  |  |  |  |  |  |  |  |  |  |  |  |  |  |  |  |  |  |  | 103 | |  |  |  |  |  |  |  |  |
|  |  |  |  | | |  | | |  | | | |  | | |  |  | |  | |  | | | | | | | |
