# Supplementary material for: A fungal endophyte induces transcription of genes encoding a redundant fungicide pathway in its host plant
Source: BMC Plant Biol. 2013 Jun 26;13:93. doi: 10.1186/1471-2229-13-93 (PMC3700885; doi:10.1186/1471-2229-13-93)
Supplement: Additional file 8 — Sequences of amplified fungal bands using either fungal genomic DNA or RNA as templates in combination with PCR primers corresponding to Taxus plant taxadiene synthase, DXR and HMGR genes. None of the amplified bands showed similarity to authentic taxadiene synthase, DXR or HMGR. Amplified bands using Taxus taxadiene synthase, DXR and HMGR primers sequences and Taxus DNA or cDNA templates were used as positive controls. [file 1471-2229-13-93-S8.doc]

**Additional file 8:** Fungal and plant PCR amplicons sequences using primers for plant taxadiene synthase, plant deoxyxylulose-5-phosphate reductoisomerase (DXR) and plant 3-hydroxymethyl glutyryl CoA reductase (HMGR)

| **Primer name** | **Primer sequence** | **Template** | **Amplicon sequence** | **Close identity** |
| --- | --- | --- | --- | --- |
| **I- Amplicons sequences from fungal genome/cDNA and plant genome/cDNA using plant taxadiene synthase primers** | | | | |
| **TSin** | GGTTTGCTCCAAATCAGGGC  TAACATTGTGGTGCCACAGA | Plant cDNA  Fig.3 lane F | AAGAGTTTGCTCCNAATCANGNNGAACCAGAGTAAAAAGAGGAAGTGGTGGTCCTGGTCCTGTCGTAATGATGAGCAGTAGCACTGGCACTAGCAAGGTGGTTTCCGAGACTTCCAGTACCATTGTGGATGATATCCCTCGACTTTCCGCCAATTATCATGGCGATCTGTGGCACCACAATGTTA | **Taxadiene synthase**  185bp |
| **TSin** | GGTTTGCTCCAAATCAGGGC TAACATTGTGGTGCCACAGA | Fungal cDNA  Fig.3 lane F | AGATTTAACATTGTGGTGCCACAGACGTATCCACCGAGGGAGATTCTCCCCAAAATATCACTAGCGTTCAAGCCCAACGGTTGATTCTGTGCACTGGATCGTCGCCAATCGACCCGCCTCTGCCATACGAGCCTGGGGGCTTGAAGCATCTACATCTTGACACTGCGCTATCGCCGACC | hypothetical protein  179bp |
| **TSin** | GGTTTGCTCCAAATCAGGGC  TAACATTGTGGTGCCACAGA | Fungal genome | AGATTTAACATTGTGGTGCCACAGACAAGTTCCCAGCTGCCGTGCTCGTCCAACGCCAGACAAATCCGCTACCCACTACCCAAGTCGGTGACCCATGCCTGGACTACTCCGTCACCGCCAACATGTCGACCATCAGCGCAAATTCATCATACCGCGCTGCCTTCATGCAGAAAGCACCGGTCGGAACAATCATCACAGCGCGCATGCTCAACGCTGCCCAAGCCAAGCTCCCAGCACTCACAGCAGACGTAGCACTCAACCAGCAATGTGGAAACCTTACCCAGCTTGCATTTACGGAAGCTGCCAACAACTTCTCGAACGGCATTGTCGCCCAGTTCACCACCGAGGGTCTGCCTGTTGGTATCAAAGCTGGTCCTGAGGTTGTAGTCATCGTCGGCGCTATTTGTGCACTCTTTTCGATTGTCTGGGTCTTCGCTGGATAATGGAGCGTAGAAGCCTGCTATTGGCAGAAGTATTGATGGGTATGGCATATCATTGTGAGCTAGCCTGGTTGTTGGAAGACTCGCTGGAGACCACTTTAGTCAGGAGCGTCCAACCGCAAACGTTGCGAGATTCCCATGACGAATCAGATAAACTGCACGTAGTCGGCGACCGCGACCTGTGTACGTAAATGAAGCTTTCTCGGGCGCAACAAGAATGGCAATATCGTGCTCTTAACACTCGAATCATTTGCACGACTACTTGCGAGCGATTAATCCTCGGACAGCTTTGGAGCGAAGTAGCGTAAGTGGTTGACGGTACGGAAATTACTGCACGAGCAACTTGATAGGATCGCTTATGTAAGCGCCATTGCAAGGTCGAGGCCCTGATTTGGAGCAAACCAATCAC | hypothetical protein  849bp |
| **TS3/in** | ATGGTGGAAGGAATCTGGCATGGC  TAACATTGTGGTGCCACAGA | Fungal genome | TTGCTCCNAATCAGGGCAAAGAGTCGCCATGAGAGCAGCAACAGACAGACGTCGAGTCTTCATGTCCGACATGTGGTACAGATCATCCGGATTCAACAGGGGGAGATATTACCTGGTGAGCTGAACCCAGGTTTGTTTAATGCTTTTCATTGTTACTCACTTTCATAGCAATGTCTGTGGCACCACAATGTTA | No Significant Similarity |
| **G1** | CAGGCCGCTGATGGTAGTTGG  GCITAIGAIACIGCGCITGGGT | Fungal genome | TCGAGTTTTTCGCAAGANCATTGNCATATCGGNACACTGNATGGTGCTCCCAAAGCACGAAAGNAATGTATCTCCTGTATCCTTTTCACCCGCTCANGATTGCACAGGNAANANATGCTACGTCNNCANTGTCCGTGGCATGGNGATAACTGAGATGGATCGAGATTGGGACTCGACCGGNCTCCACGGANGAATACTCACCAAANAGATGGGCCTGNAGAAAGCAAAGA | No significant similarity found |
| **G2** | AGGAAAGGTGCCTGAGATGCCACC  GAAIGCCATIGCIGTITCITC | Fungal genome | CTTATTGCAGGCTTTACTCCCCATTTCGTTACCCNNATGCTCNCNNANTCNCAANCNNATANACGGCTATCCNCCTGANCATATCTGTGTTATCGAACAATTGTCCTANGAACGCCTCTCCCCNNNCCCNCNGNATGACNTAANACCCGATATCTCCNANGGNAGNTGNGTGCGATATGANCTGCNTGANNAGGAAAACNACNGNGCTGAGTACAANCATGTGGATTATGATGNGNGCCTTCNNGGCATCNAGTTGGTGCTTGTGAGGAGTANTAACANCGNATAANGNNNGANACAACNTGNANNACATGGTTGNATGAAGGNACNNTCTTGTGGATGAGAAAGGAT | No significant similarity found |
| **TS12** | TAGGGAACAAGGCAATCCAC  CGTTCTTGGTAAGTAGAACT | Fungal genome | Two amplicons  (1)AGATTTAGGGAACAAGGCAATCCACAACATTACGCGGGAGCGAAGAGCGGCTATTCTAGCCGGAAGTAGCCTAGGGTTGTCGACGAACACTGTGAAAACACTCATAAGTGTTACCACACGCTGCCACCCGTGAACAATACCCTAGACACCAAACACGATGTCTCCAAAATCTACAGTCATATCAGTCATCGGCTCTCTCAACGTGGACCTTGTCACTCGCACGCCCCGTGTGCCTGTTGCAGGTGAGACTCTGACAACGGAGTCTTTCAACACAGGATTTGGCGGAAAGGGCGCGAATCAAGCCGTCGCTTGTGCGCGACTCTCGAGAACGCAAGAGCAGGCATCGGATGGCGAAGCGTCTGATGTTGAGGTTCGCATGATCGGTGCTGTAGGAGACGATGAGTTTCACGAGGGGTTCTTGAAGAGTTTGCAGGCAGATGGGTTGGACACTAAGGGTGTCAAGATATTGAAGGGTAAGAAGACGGGCGTTGCAGTCATCGTGGTTGAAACTGGAACGGGGGAGAATAGAATAATGTTTTGCCCTGGGGCGAACTACGACGTTCAGGTAGAGGATTTAGTGGCCAATGATGCCGCTGTGGTTCTATTCCAGCTGGAATTGCCGTTGCAAGTTGTGAGTCTTCCAACCTTTCGAAGCATTCGCATAGCGACATCATGCGCTAATGAACACAAGGTATTACACAACGTGAATTTGGCACGCGAGAAGGGTGCTGAGACAATCATCAACCCTGCTCCGGCTATTCCTCTTCCGGACGAGGCCTACCGCGGCCTTGGTCATCTGATTGTAAACGAGACGGAGGCTGCTATACTATCTGGCATCAGCAACCCTACATCATGGGATGAGGTCGCATCTGTCTTCATCAACCGAAGTGTTGAAAACGTGATTATCACCCTTGGCGGAGAGGTATGGCAAGTACTCAGAACTATGACGTTGGGCGCTTAACCCTTAGTAGGGCGTAAACTATCGAACAGACAGCAACATGCAAGTCACAAGCTGTTCGATCGTGCAAGCGAGGAGTGATGGGTTGATACTACACGCACTGGTGAACCTTGGGGGCCCATTCGTGCGGTGCAAATGGATCTCGCTTAAGCAAACGGAGTTGAACTTGAACTCCGCGGCG    (2)AGATTCGTTCTTGGTAAGTAGAACTGAATAGGTCTGTAAATCGAGGGGAGGGAGGCTGTGGAGGCGCGGTGAGGTGAGAGGTGAGAGACGCTGTAAACAAAGACCGCTGAACGGGGAAGGCGCGGCGTGGCTTCACTTGAGCATCGTGTTTGATGCCAAGCACCGATTGGGCAGTCACACTGTTTATCAGACCTTTATGGTTCCTACAAGAGCTTCGCGCCTGCAATTTACGTACCTCCTAGCAAGTCAAGAAGCGCGCAAATCTCTGGACCTCTCCTAGCATTCAACCCGGTGGATTGCCTTGTTCCCTAAATCACGAATTC | hypothetical protein similar to ribokinase  cell well associated RhsD protein  323bp |
| **TS12** | TAGGGAACAAGGCAATCCAC  CGTTCTTGGTAAGTAGAACT | Fungal cDNA | TTGCGCGCTTCTTGACTTGCTAGGAGGTACGTAAATTGCAGGCGCGAANCTCTTGTAGGAACCATAAAGGTCTGATAAACAGTGTGACTGCCCAATCGGTGCTTGGCATCAAACACGATGCTCAAGTGAAGCCACGCCGCGCCTTCCCCGTTCAGCGGTCTTTGTTTACAGCGTCTCTCACCTCTCACCTCACCGCGCCTCCACAGCCTCCCTCCCCTCGATTTACAGACCTATTCAGTTCTACTTACCAAGAACG | cell well associated RhsD protein  255bp |
| **TS3** | ATGGTGGAAGGAATCTGGCATGGC  GTCGCCAGCTCAAGGATACAAGCT | Fungal genome | Two amplicons  (1)AGATTATGGTGGAAGGAATCTGGCATGGCCGAGCTTAGGTGTCCTCGCGAGGGAAGAAGGACTGAAAGCCCTGTACAAGGGTTACGTAGCCAAAATACTGAGGTTCGGGCCTGGCGGTGGTGTGTTGCTGGTCGTCTATTCTGCGGTGATAGATATGCTAGGAAAGGTAGCATAAAATTCTGGCACATGACTTTGTGAGAACAAGGTCATCCTACATTTATCGTATCCAGAGGCCGTATGCGAACGTCGGAGTCGCCCTATTAAGATTTGGAATTC    (2)AGATTATGGTGGAAGGAATCTGGCATGGCAGGCGTCATATCTTCATGTGATCTACGTGGTGGCAGAGCTGGCGGAGGTTCTTCCAGGGTGGTTCGCGTCTCGTCCGGCGTGTAGTGTGGTGGAGGAGGTGTGGTATCTATGACATGCGTATTCTCAGGGACAATGGGTGCTGGCGCAGTGTACGAGCTTGTAGCATGAGCGGCTGCATCACCTGCTGGTGCTGGCGCCGTATGCGAGCTTGTATCCTTGAGCTGGCGACAATCAC | hypothetical protein  hypothetical protein |
| **TSmix5,P** | CAAACCCATGTCGAATTGAGAAG  GAAIGCCATIGCIGTITCITC | Plant genome | CTTCCGACCTTGCATTTCCTGACGAAGGAGCTATGGACGATGCTAGAAAATTTGCAGAACCATATCTTAGAGACGCACTTGCAACAAAAATCTCAACCAATACAAAACTATTCAAAGAGGTTAGTACAATCTATTTTTATTAACAAAAATATATCAATAATTCTACCAACATAATTAGGAGTAAACCTAAAGTTTTCTATTATATGTAGTTAGAAATCAAGGTTTCTCTAATATGATGGATCATTTCAGATTGAGTACGTGGTGGAGTACCCTTGGCACATGAGTATCCCACGCCTAGAAGCCAGAAGTTATATTGATTCGAATGACGACGAATAGGTATGGCAGAGGAAGACTTTATACAGGTGAGTTCAAATATAATCACATTACCCTTCAATTTTATATAGAATTTATGAGCCCTTGTTGTATCGATTGCTATCTTTTCTCAATACTGAGGTTATAATCCGTGCAACAGAATGCCATCTTTGAGTAATTCATAATGTTTAGAATTGGCAAAATTGGACTTCAATATCGTACAATCTTTGCATCAAGAGGAGTTGAAGCTTCTAACA | Taxadiene synthase  569bp |
| **TSmix5,P** | CAAACCCATGTCGAATTGAGAAG  GAAIGCCATIGCIGTITCITC | Plant cDNA | TTTAGGTGANNCTATAGAATACAGCGGCCGCGAGCTCGGGCCCCCACACGTGTGGTCTAGAGCTAGCCTAGGCTCGAGAAGCTTGTCGACGAATTCAGATTCAAACCCATGTCGAATTGAGAAGCATGGTGAACCTTTTGAGAGCTTCCGACCTCGCATTTCCCGACGAACCACTTATGGACGATGCTAAAATCTTTGCAGAAGCGTATCTTAGAGACGCACTTACAACACGCATCTCAACCAATACAAAATTATTCAAAGAGGTTAGTGCTACCGTATTTCCCCCTTTAAAATCCACATCACTAATCCAACCCAAACCCGAATTTTGTTCCTTTCTCTAATATCATAATCATTTCATTTCAGATCGAGTATGCGGTAGAGTACCCTTGGCACATGAGTATCCCGCGTCTCGAGGCGAGAAGTTATATTGATGCATACGACGATGATTATACATGGCAAAATAAGACTCTATACAGGTGGGTCTAAATATATGATCACATTAGCCAAGGATATTTCATACAAATTCTGCGAGCCTTGATGCACCAGTTATTGTCTTTCT | taxadiene synthase |
| **TSmix5,P** | CAAACCCATGTCGAATTGAGAAG  GAAIGCCATIGCIGTITCITC | Fungal cDNA | TTAGGTGANACTATAGAATACAGCGGCCGCGAGCTCGGGCCCCCACACGTGTGGTCTAGAGCTAGCCTAGGCTCGAGAAGCTTGTCGACGAATTCAGATTGTTAGAAGCTTCAACTCCTCTTGATGCAAGCTGAATACGAATATCACCAAACGAGAGAGAACCAGAGGGTCCCAAACGCCTATAAGAACAGTTATCATTCGCTAGGAAAGGTATTCTCGACACCCATTGTCTTCTCAATTCGACATGGGTTTGAATCACGAATTCTGGATCCGATACGTAACGCGTCTGCAGCATGCGTGG | aminoadipte reductase |
| **TS5** | CAAACCCATGTCGAATTGAGAAG  CAAGTTTGCATACACTCTGGAATCT | Fungal genome | TAGGTGANNCTATAGAATACAGCGGCCGCGAGCTCGGGCCCCCACACGTGTGGTCTAGAGCTAGCCTAGGCTCGAGAAGCTTGTCGACGAATTCAGATTCAAGTTTGCATACACTCTGGAATCTCTTCGATTACTACATGCATCCCGCTGGGTCGTACTTCGGCACCAAAGTTGGCAGTCGTCTGCAACATGTCGCCTACGACTACCTCGCGCGTTCTGTCTACTTGATCAACCGTTCACTGTCGTCCTTTGGTCCCTGTACTGTCGCTGTTGAGCTGCTCGATATTAATGGCACAACGATTATGAATACCACGACGACCACTCAAATTTCCCCTAACAACAGCACCCAGCTGTTCGAGGTTGAGAATATCGATAAAATCAGGGATGTAGCCTTATTGAAACTTGTACTTAAGAGCGAGGAGAAAGTCCTCTCAAGGAATGTATACCACCTCACGCCCAAACCCGACACCCTTGACTTCTCAATTCGACATGGGTTTGAATCACGAATTCTGGATCCGATACGTAACGCGTCTGCAGCATGCGTGGTACCGAGCTTTCCCTATAGTGAGTCGTATTAGAGCTTGGCGTAATCATGGTCATAGCTGTTTCCTGTGTGAAATTGTTATCCGCTCACAATTCCACACAACATACGAGCCGGAAGCATAAAGTGTAAAGCCTGGGGTGCCTAATGAGTGAGCTAACTCACATTAATTGCGTTGCGCTCACTGCCCGCTTTCCAGTCGGGAAACCTGTCGTGCCAGCTGCATTAATGAATCGGCCAACGCGCGGGGAGAGGCGGTTTGCGTATTGGGCGCTCTTCCGCTTCCTCGCTCACTGACTCGCTGCGCTCGGTCGTTCGGCTGCGGCGAGCGGTATCAGCTCACTCAAAGGNGGTAATANGGTTATCCACAGAATCGGGAAACGCGTGAGCAAAAGCCAGCAAAGGCAGGAACCGTAAAAGGCCGCGTTGCTGGCGTTTTCNT | glycosyl hydrolase |
| **TS5** | CAAACCCATGTCGAATTGAGAAG  CAAGTTTGCATACACTCTGGAATCT | Fungal cDNA | GGTGANACTATAGAATACAGCGGCCGCGAGCTCGGGCCCCCACACGTGTGGTCTAGAGCTAGCCTAGGCTCGAGAAGCTTGTCGACGAATTCAGATTCAAGTTTGCATACACTCTGGAATCTCTTCGATTACTACATGCATCCCGCTGGGTCGTACTTCGGCACCAAAGTTGGCAGTCGTCTGCAACATGTCGCCTACGGCTACCTCGCGCGTTCTGTCTACTTGATCAACCGTTCACTGTCGTCCTTTGGTCCCTGTACTGTCGCTGTTGAGCTGCTCGATATTAATGGCACAACGATTATGAATACCACGACGACCACTCAAATTTCCCCTAACAACAGCACCCAGCCGTTCGAGGTTGAGAATATCGATAAAATCAGGGATGTAGCCTTATTGAAACTTGTACTTAAGAGCGAGGAGAAAGTCCTCTCAAGGAATGTATACCACCTCACGCCCAA | glycosyl hydrolase  458bp |
| **TS1** | AGCACTGGCACTAGCAAGGT  TTCACAACCAGCTCATCTGC | Fungal genome | TTAGGTGACTATAGAATACAGCGGCCGCGAGCTCGGGCCCCCACACGTGTGGTCTAGAGCTAGCCTAGGCTCGAGAAGCTTGTCGACGAATTCAGATTCAAACCCATGTCGAATTGAGAAGATGGACGTAGGGCCAGCCGCAACAAATCAAAACCGCGCTATCTGGTTTTCCTCTGCACGTCCCGGCCAGCAAATGGTTTCGGTCGATCCCCAGTATTGGTCGGATAATATGAAACATCCTGTCTTCTTCAAAGACGCCGTCTCCGCTGCATTCAAAGAAACAGGCGCCCCGGACTTGGTACTGGAAATCGGTCCGCAACCAGTACTAGAGAGGGCTGTTCGGCGAACCATTACTGAGTTCGATGACAGCAGCCCCATGTACACTGGCTTACTCAAACGCGGTTCGGATGCAGTCACTTCCCTCTCGGGAGCCCTTGGCCTGATATGGAGCTACTTTGGGCACGAAGCTGTTGACATGGCGAGTCTCGATCGAATGCTATCTGGCCTACCACAGCCAAAGTTGGTAAAAGGTCTACCTACCTATCGTTGGCAGTATGACAAAGAGTACTGGTGGCAGAGCAGATACATGCGCAAGCAGCTTCAGTCTACTACTCCACCGACTGAGTTACTAGGGCCAGAAACTCACATGAGCGCTAGCCACGAAGCCAGATGGAGGCAATTCCTGGATCCAAAGCAGAGTCCATGGGTGTTGGACCACAAGATCGATGGAGTTGCTGTGCTTCCAGCCGCAGCATACGTCTCGATGATTGTGGCTGCTGTTCAAAACAAATATGGCGACAGTGGCATTGTTTCGATTGATGTAACCAGTCNNANNNTCCAGCAACCTGTCATCTTCGCGAGCGAGTATGCCAAGGTCGAAACAATTCTGACGATGCATGGNTTGAAAGAGANCTTTCAGCACATATCNNNTCAATTACNATCGACTTTTGTGCTGACCAGGCGAACAATGGCTTGNTTACAGCGGTAGTAGGAAGCTTCTGCGTCACTCTGGGCAA | polyketide synthase |
| **TS2** | TGTTATCGCCCTCTCGGTTTGGAA  CGCCGAATTTGTCCAGCAGATTGT | Fungal genome | ANNCTATAGAATACAGCGGCCGCGAGCTCGGGCCCCCACACGTGTGGTCTAGAGCTAGCCTAGGCTCGAGAAGCTTGTCGACGAATTCAGATTCAAACCCATGTCGAATTGAGAAGGTGAAGGCAGTGTCATCGGGTACGCATGCGGAAGAGCGGCGTTGAAGAAGTTAGGTGCTAAGAGGGGCGCAGTATGCGATTCAGGCAATGCCTAGGCACGTTGGGCTTGGCAGGTGCCTGGGTTCTGGGGGATCGTCGTGCGCCTGGACTCAAAGAGTCTGAGGGACAAGAGTGGCATAGCTAGTGGCACAACTTCTCGTCCAATTTGACGTTGCAGGATGGAAGCGTTCAACAAGACGCCGTAAACGGTTCCAGTTAGTTCCATGTTTTATGTCTCAGAACGCGAGGGTGACGGTGATTCTATTGCTCGATCCTGAAAACTATCTAGCAAAGGTGGAAACGATCCTCCATTGACGCCAAAAATTGACGCCAAAACGGAGCAAACTATATTCTCCAAAAGAAGACCTGTAATAGCAATTTTACACTCTTCCCCCGTTCAGCTCGATTCCCTGACACTGAAAATAAGATGGCGGATTGCTGCGTTCCACATTATTCAATCGCCTCTATTAAGCCTGAAAGAGAACATTTCGCTTGGAAGGAATCGGACCGGGTGGAATGCAAGAAAAGCGCATAGAGCTCCTGCTCAGATATAAGCAACAAAATGCGGCACTGTCGCCTTCCACCGCCCAGCACGATTTGGGGGCCAATGTTACACGGGCGTATAANGNTGAAACCCCCACCATACTTTAATCTAAGGCAGCACGTACCTTTAGCGTAGTTCATAAGCTGANCCCCTACGGAGCGCAGCGTTCGTTTTACCTTCCAGCAATTCGAGTCCTTCTCTTCCTTCACAGTTTGCGAACGTCCGACAGAACAATCGGGATCTTTAATCGCGANATNNNACCCCATAGTGNACTCGCANATTTTGAGTACCACCAGACTAACTANACGGT | LEA dehydrin-like protein |
| **TS4** | AATGCAGCGCTGAAGATGAATGCA  TTGGCTGTGCCCTGTTTTCCAAAC | Fungal genome | CTATAGAATACAGCGGCCGCGAGCTCGGGCCCCCACACGTGTGGTCTAGAGCTAGCCTAGGCTCGAGAAGCTTGTCGACGAATTCAGATTCAAACCCATGTCGAATTGAGAAGCTGAAGAAGAAGGACGATGTGGAAGGCACCTGGTTTGCGAGGCGGAGCCTGCATGAGGAGGTGAGCTACGAAGAGCTAGACGAAGTGCTAAGGAGAAATCATGAGAGGAATGAGATGGAGTATACGCCGGCGATCTACGATGTCAATACCCTTCTCGAATGGCACGTGGAGGAAGAGATCAGGGGCGTCAGGGATGTGAATCTCAGGAGTAAGTGTCTTTGGTCAATTATGCCCATATGCACGGTGGCCGATTGGTCCAGAGTTCGAGATATGTACTAATGCGTAACACCGCAGTAACACAGATGCATCACAGCATGCCCGCCCCTGCCCTTCTCAATTCGACATGGGTTTGAATCACGAATTCTGGATCCGATACGTAACGCGTCTGCAGCATGCGTGGTACCGAGCTTTCCCTATAGTGAGTCGTATTAGAGCTTGGCGTAATCATGGTCATAGCTGTTTCCTGTGTGAAATTGTTATCCGCTCACAATTCCACACAACATACGAGCCGGAAGCATAAAGTGTAAAGCCTGGGGTGCCTAATGAGTGAGCTAACTCACATTAATTGCGTTGCGCTCACTGCCCGCTTTCCAGTCGGGAAACCTGTCGTGCCAGCTGCATTAATGAATCGGCCAACGCGCGGGGAGAGGCGGTTTGCGTATTGGGCGCTCTTCCGCTTCCTCGCTCACTGACTCGCTGCGCTCGGTCGTTCGGCTGCGGCGAGCGGTATCAGCT | hypothetical protein |
| **FunTS1** | TGGATGCGGCACTGGATAAG  GCAGTTGGCATGGCAAGGTC | Fungal genome | CTATAGAATACAGCGGCCGCGAGCTCGGGCCCCCACACGTGTGGTCTAGAGCTAGCCTAGGCTCGAGAAGCTTGTCGACGAATTCAGATTGCAGTTGGCATGGCAAGGTCTATATGGGCGCAAGAGTACCAGAGCTGTATCAAGCACCGATAGCTTTGGAGCCATTTGAAGAGGTCAAGAAGACTGTGCAGGGTAATCCGCGATTCATATGAAGCGCCGCGTTTACGTGATGCAAGAAAATATATAAACGACACTGTACTTCTCATCCCGCTTCGTCAAA | aminoadipte reductase  280bp |
| **FunTS1** | TGGATGCGGCACTGGATAAG  GCAGTTGGCATGGCAAGGTC | Plant genome | CTATAGAATACAGCGGCCGCGAGCTCGGGCCCCCACACGTGTGGTCTAGAGCTAGCCTAGGCTCGAGAAGCTTGTCGACGAATTCAGATTACAGTTGGCATGGCAAGGTCCTGACTCCCTCCTACAAGCTCACATGCTGCAATGCACAGAGCAGGCCTGACGCGCTTACCTCCTGCTAGAAGTGAATACCTCATTGATTCATGTATTTTTTCAGGATATTCAAGTGGGATTGCCTTATCCAGTGCCGCATCCAAATCACGAATTCTGGATCCGATACGTAACGCGTCTGCAGCATGCGTGGTACCGAGCTTTCCCTATAGTGAGTCGTATTAGAGC | geranylgeranyl diphosphate synthase  336bp |
| **GSP** | GSP1  CAGGCCGCTGATGGTAGTTGG  GSP2  AGGAAAGGTGCCTGAGATGCCACC | Fungal cDNA | TTAGGTGANACTATAGAATACAGCGGCCGCGAGCTCGGGCCCCCACACGTGTGGTCTAGAGCTAGCCTAGGCTCGAGAAGCTTGTCGACGAATTCAGATTAGGAAAGGTGCCTGAGATGCCACCTCGTCGACTAGAACGTCCGGAAGACTAGATCCCAGCCCTTTACCTTTCTATGGCCCCATTTCGCATAGACAAAGTCCGGGGAACCGACCTGCTGGGCTAGATCGAACAGCACCAAGACGTGATCAGACACGACCACCCGAAAGTCTTCGAAGCTTTCAAGAAATTGTTTTCGATTACCACCTACAAGCAGCCACTTGTCTAAAAGTAACCTTAGCGACTGCTTACCTTGAAAACCTACGCTGTGGAAGATAGAGGCGATGGGGTTTTTTCAATAAAGATCTCATGTGTTATTCAACACACCTAAACACAGCAACGAGAGACACGCAGCTACATGCCA | aminoadipte reductase  461bp |
| **II- Amplicons sequences from fungal genome using plant DXR primers** | | | | |
| **DxR** | TGGGCTCTTTCGCGCACAAACACCACAGGCG TGAACGGTCCTGGATTCATACATACTTCCTGCG | Fungal genome | CTCGGNNCCNCTAGTAACGGCCGCCAGTGTGCTGGAATTCGCCCTTTGGGCTCTTTCGCGCACAAACACCACAGGCGCATATTCCGCAACCAGAAACCGCTGAGCTGTCGTCTTTGAGCCATCCATCGGAGGATATGAAGGTGTCTTCACCCACTGGCGCCCACATGCGCGAAGCAGATATTGCAGAGGCAATGAAAAAGAGTCCAAACGAAGAGCACCCGGA | hypothetical protein 223bp  or  photosystem II protein D1 Max score [34.7](http://blast.ncbi.nlm.nih.gov/Blast.cgi#18996285) |
| **F1** | CAGCTTAAGCTATTAAGCTAAAAC  GTGAGCTACATACACTTACG | Fungal genome | Two amplicons  (1)TCGGNTCCNCTAGTAACGGCCGCCAGTGTGCTGGAATTCGCCCTTAACAGGATTAGATACCCGGGTAGTCCACGCTGTAAACGGTGGGTACTAGGTGTGGGGTCCATTCCACGGGTTCCGTGCCGTAGCTAACGCTTTAAGTACCCCGCCTGGGGAGTACGGCCGCAAGGCTAAAACTCAAAGGAATTGACGGGGCCCCGCACAAGCGGCGGAGCATGCGG  (2)TAACGGCCGCCAGTGTGCTGGAATTCGCCCTTGCTGACTGGCGATTACTATCGATTCCCCAATGGCGAGACTGGCGGAGTTGGTGTTGAAGGCGGCAAAGGTAGAGAACACATCCGGACTTCCGTTGCCAAACGCTAGGAAGGTAACTCCTGCCATGCTCTCGCTCATACCCAATAGGTTGGCGATGGTGCTCAGGTTGACGCAAAAGAAGTCGCTCGCAGCGATGCCGATGGTGCTGAAGAGAAGCGCAAGCCATAACACGAGAATAGTGAATGCGACCGGTTTCGCATG | conserved hypothetical protein  221bp  sodium/calcium exchanger protein  291bp |
| **F2** | TGTATGGTTACTACGCTTCTTCC  TAGTACCAACTAAAAAAAGAGCTTTCG | Fungal genome | Three amplicons (1)TAGTAACGGCCGCCAGTGTGCTGGAATTCGCCCTTGCGGCATGCTTAACACATGCAAGTCGTAAAAGTGGCGTGAGATGCAAGGTAGCCTGCTCCCGTTCTCTCCAGCGAAGTAGAAATGACGCAAAGAAATGATGAGGCATCGTCCCGCCGTTGGAGACCGCCGTGTCCATCTTGGACTGCTGAGGATATCGAAAGTCAGGTCGAAAATGCCCGAACAATCATGTACAGAAGGTATTTGCAATGCCCTTCACAAGACCACAAATCCCGTATAATGTGATGATATCAAG  (2)AACGGCCGCCAGTGTGCTGGAATTCGCCCTTGCTGACTGGCGATTACTATCGATTCCCCAATGGCGAGACTGGCGGAGTTGGTGTTGAAGGCGGCAAAGGTAGAGAACACATCCGGACTTCCGTTGCCAAACGCTAGGAAGGTAACTCCTGCCATGCTCCCGCTCATACCCAATAGGTTGGCGATGGTGCTCAGGTTGACGCAAAAGAAGTCGCTCGCAGCGATGCCGATGGTGCTGAAGAGAAGCGCAAGCCATAACACGAGAATAGTGAATGCGACCGGTTTCGC  (3)CNANNGNGNTGGAATTCGCCCTTGNNGNATGCTTAACACATGCAAGTCGCGGTCGCACCGNGCAAGACTTCTTCCGTCCTGNCCATGANCCGCACGGNTGGNGAACGTCNNGNNCGCACCACGTTCCGCTTGNGCCCACTCATCCTTTTCATTCTTTATTTCCTCGACCGTCCTTGCTTGCGTCTATCTCCGCCTCGCTTCGCTGCGATCAATCGTCATANGAAGGGTTTCGCTGACTGGTATGCANGTCAACAATGNGATCATCGCGGGTAGCGACGAGAACGAGTTCTAGACGCGCGGTCGCGGCTGCTGAGGGACCAAGGNNGCCACGTGGGTGAAGGACGAGGGACCTGNATGCACGATAAGCGCTTANGGAGGANAGATGTACGACGCCCTATACCACATATAGACGGNAAATGTAATGCACAAGTCAAGGNAT | pyruvate carboxylase subunit A  296bp  sodium/calcium exchanger protein  287bp  No Significant Similarity  439bp |
| **III- Amplicons sequences from fungal and plant cDNA using plant 3-hydroxymethyl glutyryl CoA reductase primers** | | | | |
| PlantHMGR2 | TCCCTGTGGGTGTTGCAGGGC  GGGCTCCCGTTGTTAGGTT | Plant cDNA | GGTGANNCTATAGAATACAGCGGCCGCGAGCTCGGGCCCCCACACGTGTGGTCTAGAGCTAGCCTAGGCTCGAGAAGCTTGTCGACGAATTCAGATTCCTCCTATTCTCTTGAATCCTCACTTGGCGACTGCAAGAGAGCCGCCTTTGTGAGGAGGAGTGCTTTAGAGCTCATGACTGGGAGATCTCTTGATGGGCTGCCCTTGGAGGGATTTGATTATCAGTCCATTCTCGGGCAGTGCTGTGAGATGCCCATTGGGTATGTACAGA**TCCCTGTGGGTGTTGCAGGGC**CTTTGCTACTCAATGGGTTTGAGTACATGGTGCCCATGGCAACCACAGAAGGGTGCCTGGTGGCCAGCACCAACAGAGGTTGCAAGGCCATACATATGTGTGGTGGTGCTACAAGCATTCTTCTCAGAGATGGCATGACTA**GGGCTCCCGTTGTTAGGTT**TCAGAGTGCCAAAAGGGCTGCAGATCTTAAATACTATATTGAGGATCCCACAAACGCTGAGAATCTGTCGCACATCTTCAACAGGTTGGTGTTTATATATATTTTTAATGGCATATCTTATCTTGCATGCATCATGGGTCATCGTACAGTTTTATTAATCCATGCTTTTGGCTGATAAGGTTTTGGGTGCCCGCTATTTCAACAGGACAACCAGATTTGGCAGGCTGCAAGGCATGCAATGTGCAATTGCAGGCAAAAATTTGTACATGAGATTTTGCTGTTTCACTGGAGATGCCATGGGGATGAATATGGTGTCGAAGGGTGTTCAGAATGTCTTAGATTATCTTCAGACTGTGTTCCCCGACAT | *Taxus x media* 3-hydroxymethyl glutyryl CoA reductase  Amplified fragment  181bp  No detectable amplicon from fungal genomic DNA or cDNA templates |
