# Supplementary material for: A fungal endophyte induces transcription of genes encoding a redundant fungicide pathway in its host plant
Source: BMC Plant Biol. 2013 Jun 26;13:93. doi: 10.1186/1471-2229-13-93 (PMC3700885; doi:10.1186/1471-2229-13-93)
Supplement: Additional file 9 — Additional methodology details, including: experimental design, error and replicates, isolation of endophytic fungi, fungal genotyping, terminal restriction fragment length polymorphism (tRFLP), Taxol quantification, callus and suspension culture initiation protocol, RT-PCR, quantitative real-time PCR, and identification of taxadiene synthase (TS) enzyme by Western blot analysis. [file 1471-2229-13-93-S9.doc]

**Additional Methods 1**

**General Experimental Design, Error and Replicates**

Fungicide was applied to young plants and independently replicated using old wood. For young plant treatments, three intact seedlings were injected with fungicide, with another three injected with buffer. For subsequent sampling, large stem sections (~15 cm long) without needles were harvested from each plant to overcome micro-scale variation. Each tissue sample was divided into small sections and then pooled. Hence there were two biological replicates, each representing pooled tissue subsamples. The experiment was independently replicated using old *Taxus* wood. For old wood, two randomized pools of 4-6 cultured wood pieces were treated with either fungicide, herbicide or buffer as indicated. Hence, for each old wood treatment, there were two biological replicates, each from tissues that were independently treated and then pooled.

For Taxol quantification performed using the Taxol immunoassay, each biological replicate was assayed in triplicate (3 technical replicates). The biological replicate number is noted in each experiment.

For all experiments, the error bar shown is the standard error of the mean (SEM) of the biological replicates. Statistical comparison of means, along with all raw data, is presented in Table S1 with each statistical test noted. Statistical analysis was conducted using In Stat 3.0 (GraphPad Software, USA).

**Endophyte Natural Variation Sampling in *Taxus***

For natural variation sampling, 33 intact trees representing diverse *Taxus* species, of different ages, were sampled during different seasons over a two-year period, to maximize the diversity of the test population. A single plant sample (~5-6 cm stem, without needles) was taken from each plant and was divided into 4-8 sections; a random pool was used for quantification of plant Taxol and endophytic fungal quantity, with the remaining pool used to culture endophytic fungus for subsequent fungal Taxol quantification. Endophytic fungi within the plant were tracked using fungal strain specific DNA fingerprinting in combination with semi-quantitative tRFLP (see below).

**Materials and Reagents**

The following reagents were from Sigma (USA), including Taxol (# [T7402](http://www.sigmaaldrich.com/catalog/ProductDetail.do?N4=T7402|SIGMA&N5=SEARCH_CONCAT_PNO|BRAND_KEY&F=SPEC)); fungal nutrient media: yeast-peptone-dextrose (YPD) (# Y1375) and potato-dextrose-agar (PDA) (# [70139](http://www.sigmaaldrich.com/catalog/ProductDetail.do?N4=70139|FLUKA&N5=SEARCH_CONCAT_PNO|BRAND_KEY&F=SPEC)). Maxim-XL fungicide was from Syngenta. Isoxaben was from Dow Agrochemicals. All solvents used for extraction, TLC and HPLC, were HPLC grade and obtained from Fisher Scientific. *Taxus* plant tissue samples were collected from the Arboretum and the University of Guelph Main Campus. *T. x media* plantlets were from Mori Nurseries, Niagara-on-the Lake, ON, Canada.

**Strain**

The fungal strain described in this paper, *Paraconiothyrium* SSM001, has been deposited at ATCC (Accession # ATCC MYA-4697).

**Isolation of endophytic fungi**

Endophytic fungi were cultured from old branches of *Taxus media* plants cultivated on the University of Guelph Main Campus and Arboretum (Guelph, Canada). Fungi were isolated from fresh plant tissue immediately after harvesting. Tree branches were cut into 1 cm long X 0.5 cm diameter pieces and sterilized as follows: 2.5% sodium hypochlorite solution for ten minutes; 70 % ethanol for five minutes; washed in sterile double distilled water three times. The outer bark and the phloem were removed and the wood was further sterilized using 70% ethanol for five minutes, followed by flaming and washing three times with sterile double distilled water. A control tissue (newly developed branch pieces from *T. media*) was used as a negative control for the absence of any contaminated fungal spores during the sterilization process and culturing. Each piece of tissue was then cut into smaller pieces (2 mm X 5 mm) and cultured on PDA media in Petri plates at 25^o^C in the dark until the first fungal growth. Hyphal tips were then consecutively transferred twice onto fresh PDA media to ensure fungal culture purity.

**Fungal genotyping**

For taxonomic classification and to ensure consistency between experiments, every fungal culture was genotyped by PCR and DNA sequencing of the internal transcribed spacer regions (ITS) of 18s rDNA to confirm both strain identity and purity. The fungal mycelia were disrupted by mortar and pestle under liquid nitrogen and then genomic DNA was isolated using the Plant DNeasy Kit (Qiagen, Germany) with additional adsorption of the DNA onto a silica membrane, washing and centrifugation as described in the manual instructions . Fifty nanograms of the DNeasy-purified genomic DNA were added into a 20 ul PCR reaction, with 1.5 mM MgCl_2_, 0.2 mM dNTPs, 1X buffer (Green GoTaq Flexi Buffer, Promega, USA, # M8911), 0.5U *Taq* DNA polymerase (New England BioLabs, USA), and 0.2 µM of each previously published universal primer set specific for fungal ITS1 and ITS4 . The following conditions were used for PCR amplification: 94˚C for 2 minutes, followed by 35 cycles of: 94˚C for 45 seconds, 45˚C for 1 minute and 72˚C for 2 minutes; with a final extension cycle of 4 minutes at 72˚C. The amplified fragment was purified using an Illustra GFX 96 PCR Purification kit (GE Healthcare, USA), cloned and sequenced. Each resulting sequence was then aligned with the sequence of *Paraconiothyrium* strain SSM001 using the Align-BLAST search tool.

**Terminal restriction fragment length polymorphism (tRFLP)**

All fungal and plant genomic DNA was isolated using DNeasy (Qiagen). Specific fluorescent labelled fungal 18S primers were used for amplification. The primer sequences were nu-SSU-0817 5`-TTAGCATGGAATAATRRAATAGGA-3’ and nu-SSU-1536 5’-ATTGCAATGCYCTATCCCCA-3’ which amplify a 762 bp fragment . The reaction cocktail consisted of 70ng of genomic DNA, 0.5 U of Taq DNA Polymerase (New England BioLabs Inc., MA, USA), 1X Buffer (Colourless GoTaq Flexi Buffer, Promega, USA, # M8911), 200 μM dNTPs and 100 pmol of each primer in a final volume of 20 μL. The PCR conditions were: denaturation at 94°C for 2 min, followed by 35 cycles of denaturation at 94°C for 45s, annealing at 45°C for 30 s, extension at 72°C for 2 min and a final extension for 5 min at 72°C in a PTC200 thermocycler (MJ Research). A 20 µl PCR aliquot was restricted with 5U of HaeIII (New England BioLabs Inc., MA, USA) in a final volume of 20 μL. The digested PCR product was precipitated with 0.1 v/v 3 M sodium acetate; pH 4.8 at 25°C and 2 volumes of absolute ethanol, then pelleted at 15,000 rpm for 10 min and washed twice with 70% ethanol. The washed pellet was dissolved in 6 μL Tris–EDTA buffer (100 mM Tris–Cl; pH 7.6 and 10 mM EDTA; pH 8.0). Samples were submitted to the University of Guelph Genomics Facility for tRFLP separation analysis using a capillary electrophoresis ABI prism 310 DNA Sequencer (PE Applied Biosystems, Canada). tRFLP data was analyzed using ABI Prism 310 Collection version 2.0 and Peakscanner Analysis Software (version 1.0).

**Taxol quantification**

Taxol was verified and quantified either by a competitive immunoassay procedure , TLC spot densitometry or by HPLC-UV.

*1. Taxol quantification by densitometry*

Taxol was applied on standard TLC plates (10 X 20 cm, Fisher Scientific #4861-320) along with three different concentrations of standard Taxol (2, 4, 16 μg). The TLC plates were then developed in solvent system A (chloroform: methanol 5.0:0.5). For compound visualization, the TLC plates were dipped in 0.5% vanilline-sulphuric acid reagent for one minute, dried and scanned using CanoScan LiDE 600F scanner and saved in Tiff format. Plate colours were then enhanced and Taxol spot densitometries were performed with a FluorChem 8800 with AlphaEaseFC FluorChem 8900 software (Alpha Innotech Corp). The process was validated by comparing ten different concentrations of Taxol standard on ten different TLC plates. The process can be used to measure Taxol down to 1-μg but not less.

*2. Taxol competitive immunoassay*

For immunoassay-based quantification , a kit (# TA02, Hawaii Biotechnology Group, Inc., USA) in conjunction with a commercial Taxol monoclonal antibody (#SC-69899, Santa Cruz Biotechnology Inc., USA) was employed. Manufacturer’s instructions were followed. Briefly, each 96-well microtiter plate, pre-coated with Taxol, was blocked with 1% BSA in PBS buffer. In the absence of exogenous Taxol, the Taxol monoclonal antibody fully bound this solid phase-linked Taxol, and this binding was detected by a second goat antibody conjugated to alkaline phosphatase (AP) which, upon binding, cleaved its colorimetric substrate, p-nitrophenyl phosphate. The change in absorbance was measured using a Spectramax Plus 384 instrument (Molecular Devices, USA) using dual wavelengths at 605 nm and 450 nm. In the presence of either a Taxol standard or sample, aqueous Taxol competed with solid phase-linked Taxol for binding to the Taxol antibody, causing a decrease in absorbance at 450 nm. The amount of Taxol in each sample was then calculated using a Taxol inhibition curve. The standard inhibition curve of authentic Taxol was generated by adding Taxol (90, 30, 10, 3.3, 1.1, 0.37, 0.12, and 0.0 ng/mL) to non-Taxol-producing *Fusarium* liquid extract for quantitative measurements . The use of *Fusarium* extract was used to subtract against any fungal cross-reactivity with the Taxol antibody. For sample reads, blank well values were first subtracted, these being wells receiving all reagents except a source of exogenous Taxol.

*3. Taxol quantification in intact plants and wood by HPLC-UV*

To quantify the amounts of Taxol within different samples, a set of serial dilutions of standard Taxol (0, 20, 40, 60, 80, 100 and 120 µg/ mL) was used to plot a linear calibration curve. The peak area of each sample injected was measured by a UV detector (Abs 233nm) and then factored against the calibration curve generated from injecting different Taxol concentrations. For each treatment, two sets of plant samples were prepared, one set was injected directly into HPLC and the other set was spiked with both 200 ng standards Taxol and baccatin III prior to injection. The spiked peak area was then calibrated and subtracted from the spiked amount of standard Taxol.

**Callus and suspension culture initiation protocol**

Needles and very young stem sections of *T. x media* were surface sterilized with 3% Clorox solution containing two drops of Tween 20 for 20 min with shaking. The sterilized explants were dipped into 70% ethanol for approximately 5 min and then washed five times with sterile deionized H_2_O. The tissues were then cut into small explants (0.5-1 cm^3^) prior to being placed onto B5CA culture medium. B5CA consists of Gamborg's B5 medium supplemented with 0.2% casamino acids (CA), 1% sucrose, 1 mg/L 2,4-D, 0.75% agar, with pH adjusted to 5.5 prior to autoclaving . Cultures were initiated in the dark at 25^o^C. The callus culture were subcultured every 1-2 months interval after the removal of explants. Suspension cultures were initiated from two-month old calli by placing approximately 2 g tissues in 1 L Erlenmeyer flasks containing 100 mL B5CA medium at 25^o^C in dark and shaking at 100 rpm for 2 weeks. The fungal liquid medium was filter sterilized and added to the suspension culture. The suspension cultures were continually grown for another one month prior to sampling.

**Taxane analysis in plant cell cultures**

*Taxus* suspension cultures were filtered and the liquid medium was extracted with 50 mL chloroform: methanol (10: 1) three times. The filtered, washed organic layer was then evaporated at 45^o^C under reduced pressure. The residue was then subjected to HPLC analysis against standard Taxol.

**RT-PCR**

RNA was prepared using RNeasy Mini Kit (QIAGEN, Mississauga, ON, Canada). Contaminating genomic DNA was eliminated by loading RNase-free DNaseI onto the filter column (QIAGEN). Nucleic acid extraction was performed according to manufacturer's instructions. First-strand cDNA was synthesized using oligo-dT primers with M-MuLV reverse transcriptase according to the manufacturer's instructions (Fermentas, USA). Two hundred nanograms of total RNA were used for cDNA synthesis; the synthesized cDNA was subsequently diluted with nuclease-free water (QIAGEN) to 100 ng/µL. Semi-quantitative RT-PCR amplification mixtures (20 μL) contained 100 ng template cDNA, 1.5 mM MgCl_2_, 0.2 mM dNTPs, 1X buffer (Green GoTaq Flexi Buffer, Promega, USA, # M8911), 0.5 U *Taq* DNA polymerase (New England BioLabs, USA), and 0.2 µM of each primer set. RT-PCR was performed using the following primers: for plant 3- hydroxy3-methyl glutaryl CoA reductase gene, primers PlantHMGRF3 (5’-ACTGTATTACCATGATGG-3’) and PlantHMGRR3 (5’-TGTTGTATTTCATGTGAC-3’) were used. For 3-deoxy xylulose-5-phosphate reductoisomerase (DXR), primers PlantDXRF4 (5’-AGGTGGAACCATGACTGG-3’) and PlantDXRR4 (5’-TGCAGCATACTTTCTGGCCC-3’) were used. For taxadiene synthase, primers TSinF (5’-GGTTTGCTCCAAATCAGGGC-3’) and TSinR (5’-TAACATTGTGGTGCCACAGA-3’) were used. The following conditions were used for PCR amplification: 94˚C for 2 min, followed by 40 cycles of, 94˚C for 45 s, 56.6˚C for 1 min and 72˚C for 2 min, and then a final extension cycle of one min at 72˚C in a PTC200 thermocycler (MJ Research). The produced amplicons from the PCR were applied onto a 2% agarose gel stained with ethidium bromide. The gene expression was analyzed by subtracting each band from a control band followed by dividing on a reference band. For fungal quantification, fungal 18S rRNA specific primers were used 18S rRNA-RtF (5’-GGCATCAGTATTCAGTTGTC-3’) and 18S rRNA-RtR (5’-GTTAAGACTACGACGGTATC-3’) with 28 PCR cycles. *Taxus* 18S rRNA was used as an internal standard for normalization using the following primers: Tax18SF2 (5’-TTTTCCCTTTGCAATGCC-3’) and Tax18SR2 (5’-TCGCCCTTGTAATAACCCG-3’). The same primers were also used for plant 18S quantification using 28 cycles.

**Quantitative real-time PCR**

RNA was isolated and PCR reaction efficiencies were determined by a series of ten-fold dilutions using fungal 18S rRNA specific primers , 18S rRNA-RtF (5’-GGCATCAGTATTCAGTTGTC-3’) and 18S rRNA-RtR (5’-GTTAAGACTACGACGGTATC-3’). Amplification conditions were as follows: 95˚C for 10 min, followed by 40 cycles of: denaturation, 95˚C for 15 s; annealing 56.6˚C for 30 s; extension at 72˚C for 1 min. The specificity of the reaction is given by the detection of the *T_m_*s of the amplification products immediately after the last reaction cycle. Results were analyzed with the melting curve StepOne analysis software (Applied BioSystems) . The relative expression ratio of the fungal gene was analyzed based on real-time PCR efficiency and the crossing point differences of the samples versus the plant 18S rRNA (Tax 18S), Tax18SF2 (5’-TTTTCCCTTTGCAATGCC-3’) and Tax18SR2 (5’-TCGCCCTTGTAATAACCCG-3’). The results were verified using REST (Relative Expression Software Tool) .

**Identification of taxadiene synthase (TS) enzyme by Western blot analysis**

*Protein Extraction*

One hundred mg of frozen tissue was ground in 150 µL extraction buffer [50 mM Tris-HCl (Fisher Scientific) pH 7.5, 150 mM NaCl, 10 mM MgCl_2_ (Fisher Scientific), 1% Nonidet-P40 (NP-40) (BDH Ltd., Poole, England)] supplemented with complete mini EDTA-free protease inhibitor cocktail tablets (Roche Diagnostics, Penzberg, Germany) for extracting fungal tissues or extraction buffer (0.125 M Tris-HCI, pH 6.8, containing 22.5% β-mercaptoethanol, 9% SDS and 22.5% glycerol) supplemented with 1% glycine to reduce protein denaturation for extracting *Taxus* stem tissues. Tubes were centrifuged at 13000 x *g* for 25 min at 4ºC after which the supernatants were transferred to new tubes and stored at -80ºC prior to use.

*Protein Quantification*

Protein samples were diluted either 10x, or 100x in McIlvaine’s buffer (0.021M citric acid, 0.058M NaH_2_PO_4_, 0.02% (w/v) NaN_3_, pH 5.6) for quantification using the Bio-Rad Protein Assay (Bio-Rad Laboratories, Hercules, CA, USA) according to the manufacturer’s instructions. The standard curve used contained 0, 25, 125, 250, 500, and 750 µg/mL of bovine serum albumin diluted in McIlvaine’s buffer according to the manufacturer’s instructions (Pierce, Rockford, IL, USA).

*SDS-PAGE and Staining*

Proteins were separated using sodium dodecyl sulfate polyacrylamide gel electrophoresis (SDS-PAGE) , under reducing conditions. The Mini-Protean II dual slab cell (Bio-Rad) apparatus was used for casting and running of the gels. To prepare two 1.5 mm thick gels 20 mL of separating gel solution [10.3% monomer present (T)] and 8 mL of stacking gel (3.85% T) solution were made. The separating gel mixture contained 6.67 mL acrylamide stock solution [30% (w/v) acrylamide, 0.8% (w/v) N,N’-methylene-bis-acrylamide (Bio-Rad)], 2.5 mL separating gel buffer (3M Trizma base, pH 8.8), 0.2 mL 10% (w/v) SDS, 9.62 mL ddH_2_O, 1 mL 1.5% (w/v) (NH_4_)_2_S_2_O_8_, and 10 µL TEMED (Bio-Rad). The stacking gel mixture contained 1 mL acrylamide stock solution, 2 mL stacking gel buffer (0.5 M Trizma base, pH 6.8), 0.08 mL 10% (w/v) SDS, 4.52 mL ddH_2_O, 0.4 mL 1.5% (w/v) (NH_4_)_2_S_2_O_8_, and 6 µL TEMED.

Protein samples were prepared for separation by diluting to the appropriate concentrations using the same extraction buffer prior to adding an appropriate amount of 5x SDS loading buffer [250 mM Tris-HCl pH 6.8, 10% SDS, 50% (v/v) glycerol, 25% (v/v) β-mercaptoethanol, 0.005% (w/v) Bromophenol blue]; samples were boiled for 5 min prior to loading. Between lanes, protein was loaded in equal amounts. The gels were run at 110V until the pre-stained molecular mass markers (Fermentas) were properly separated (1.25-1.5h). Gels were then immunoblotted as described below.

*Western Blotting*

For Western blotting , gels, filter paper, and nitrocellulose membrane (Bio-Rad) were gently agitated for 15 min in transfer buffer [48 mM Tris, 39 mM glycine, 1.3 mM SDS, 20 % (v/v) methanol]. Blot transfers were performed using a Mini Trans-Blot transfer cell (Bio-Rad) according to the manufacturer’s instructions. Gels were transferred at 100V for 1 h at room temperature. To confirm equal loading of samples, protein membranes were stained with Ponceau S prior to immunoblotting . Briefly, the membranes were rinsed in ddH_2_O and placed into 1x Ponceau S [0.1% (w/v) Ponceau S, 5% (v/v) acetic acid – prepared from a 10x stock] for 5 min with gentle agitation. Destaining was performed in ddH_2_O; membranes were scanned and adjusted using Adobe Photoshop 6.0 (Adobe Systems Inc., Ottawa, ON). Membranes were blocked in TTBS (0.5M NaCl, 0.02M Tris pH 7.5, 0.1% Tween-20) containing 5% (w/v) powdered skim milk for 30 min with shaking. Membranes were then submerged into the primary antibody solution (5% w/v powdered skim milk in TTBS containing a 1: 2000 dilution of polyclonal anti-TS antibody for 1 hour. The membranes were washed for 5 min in TTBS and transferred to the secondary antibody solution [5% w/v powdered skim milk in TTBS containing horseradish peroxidise (HRP)-conjugated anti-rabbit antibody (diluted 1:10000)] for 30 min (Jackson ImmunoResearch Laboratories, Inc., West Grove, PA). Blot membranes were then rinsed with TTBS, followed by washing three times in 25 mL TTBS for 15 min with agitation.

ECL Plus Western Blotting Detection Reagents (GE Healthcare, Little Chalfont, UK) were used according to the manufacturer’s instructions for chemiluminescent antigen detection. Membranes were used to expose CL-XPosure^Tm^ Film (Thermo Scientific, Rockford, IL, USA), and a Konica Minolta SRX-101A Tabletop X-Ray Film Processor (Konica Minolta Medical Imaging USA Inc., Wayne, NJ, USA) was used for film processing. Films were scanned and the contrast and brightness of the resulting images were adjusted with Adobe Photoshop 6.0 (Adobe Systems Inc., San Jose, CA, USA).

**References**

1. Omar AP-V, María JY-M, Dionicio A-R, David C-T, Silvia EG-D: **Fungi associated to *Eucalyptus*, *Eucalyptus grandis* Hill: Maid**. *Agrociencia* 2005, **39**(3):311-318.

2. Haugland RA, Brinkman N, Vesper SJ: **Evaluation of rapid DNA extraction methods for the quantitative detection of fungi using real-time PCR analysis**. *J Microbiol Meth* 2002, **50**(3):319-323.

3. Borneman J, Hartin RJ: **PCR primers that amplify fungal rRNA genes from environmental samples**. *Appl Environ Microbiol* 2000, **66**(10):4356-4360.

4. Grothaus PG, Raybould TJG, Bignami GS, Lazo CB, Brynes JB: **An enzyme immunoassay for the determination of Taxol and taxanes in *Taxus* sp. tissues and human plasma**. *J Immunol Methods* 1993, **158**(1):5-15.

5. Ruiz-Sanchez J, Flores-Bustamante ZR, Dendooven L, Favela-Torres E, Soca-Chafre G, Galindez-Mayer J, Flores-Cotera LB: **A comparative study of Taxol production in liquid and solid-state fermentation with *Nigrospora* sp. a fungus isolated from *Taxus globosa***. *J Appl Microbiol* 2010, **109**(6):2144-2150.

6. Jirovetz L, Nikiforov A, Buchbauer G, Braun D: **Correlation of TLC/densitometry-data with capillary GC-data of some main volatile components of essential oils**. *Microchim Acta* 1989, **99**(1):1-6.

7. Gangadevi V, Muthumary J: **A simple and rapid method for the determination of Taxol produced by fungal endophytes from medicinal plants using high performance thin layer chromatography**. *Chin J Chromatogr* 2008, **26**(1):50-55.

8. Soliman SSM, Tsao R, Raizada MN: **Chemical inhibitors suggest endophytic fungal paclitaxel is derived from both mevalonate and non-mevalonate-like pathways**. *J Nat Prod (in press)* 2011.

9. Gamborg O, Miller R, Ojima K: **Nutrient requirements of suspension cultures of soybean root cells**. *Exp Cell Res* 1968, **50**:151-158.

10. Gibson D, Ketchum R, Vance N, Christen A: **Initiation and growth of cell lines of *Taxus brevifolia* (Pacific yew)**. *Plant Cell Rep* 1993, **12**(9):479-482.

11. Fang W, Bidochka MJ: **Expression of genes involved in germination, conidiogenesis and pathogenesis in *Metarhizium anisopliae* using quantitative real-time RT-PCR**. *Mycol Res* 2006, **110**:1165-1171.

12. Pfaffl MW, Horgan GW, Dempfle L: **Relative expression software tool (REST©) for group-wise comparison and statistical analysis of relative expression results in real-time PCR**. *Nucl Acids Res* 2002, **30**(9):36-46.

13. Favaloro J, Treisman R, Kamen R: **Transcription maps of polyoma virus-specific RNA: analysis by two-dimensional nuclease S1 gel mapping**. *Methods Enzymol* 1980, **65**(1):718-749.

14. Senatore A, Trobacher CP, Greenwood JS: **Ricinosomes predict programmed cell death leading to anther dehiscence in tomato**. *Plant Physiol* 2009, **149**(2):775-790.

15. Roberts DR, Flinn BS, Webb DT, Webster FB, Sutton BCS: **Abscisic acid and indole-3-butyric acid regulation of maturation and accumulation of storage proteins in somatic embryos of interior spruce**. *Physiol Plant* 1990, **78**(3):355-360.

16. Konno K, Yasui H, Hirayama C, Shinbo H: **Glycine protects against strong protein-denaturing activity of oleuropein, a phenolic compound in privet leaves**. *J Chem Ecol* 1998, **24**(4):735-751.

17. Bradford M: **A rapid and sensitive method for the quantitation of microgram quantities of protein utilizing the principle of protein-dye binding**. *Anal Biochem* 1976, **72**(1-2):248-254.

18. Laemmli U: **Cleavage of structural proteins during the assembly of the head of bacteriophage T4**. *Nature* 1970, **227**(5259):680-685.

19. Kyhse-Andersen J: **Electroblotting of multiple gels: a simple apparatus without buffer tank for rapid transfer of proteins from polyacrylamide to nitrocellulose**. *J Biochem Biophys Methods* 1984, **10**(3-4):203-209.

20. Towbin H, Staehelin T, Gordon J: **Electrophoretic transfer of proteins from polyacrylamide gels to nitrocellulose sheets: Procedure and some applications**. *Proc Natl Acad Sci USA* 1979, **76**(9):4350-4354.

21. Salinovich O, Montelaro R: **Reversible staining and peptide mapping of proteins transferred to nitrocellulose after separation by sodium dodecylsulfate-polyacrylamide gel electrophoresis**. *Anal Biochem* 1986, **156**(2):341-347.
