# Supplementary material for: A fungal endophyte induces transcription of genes encoding a redundant fungicide pathway in its host plant
Source: BMC Plant Biol. 2013 Jun 26;13:93. doi: 10.1186/1471-2229-13-93 (PMC3700885; doi:10.1186/1471-2229-13-93)
Supplement: Supplementary file 4 [file 1471-2229-13-93-S1.doc]

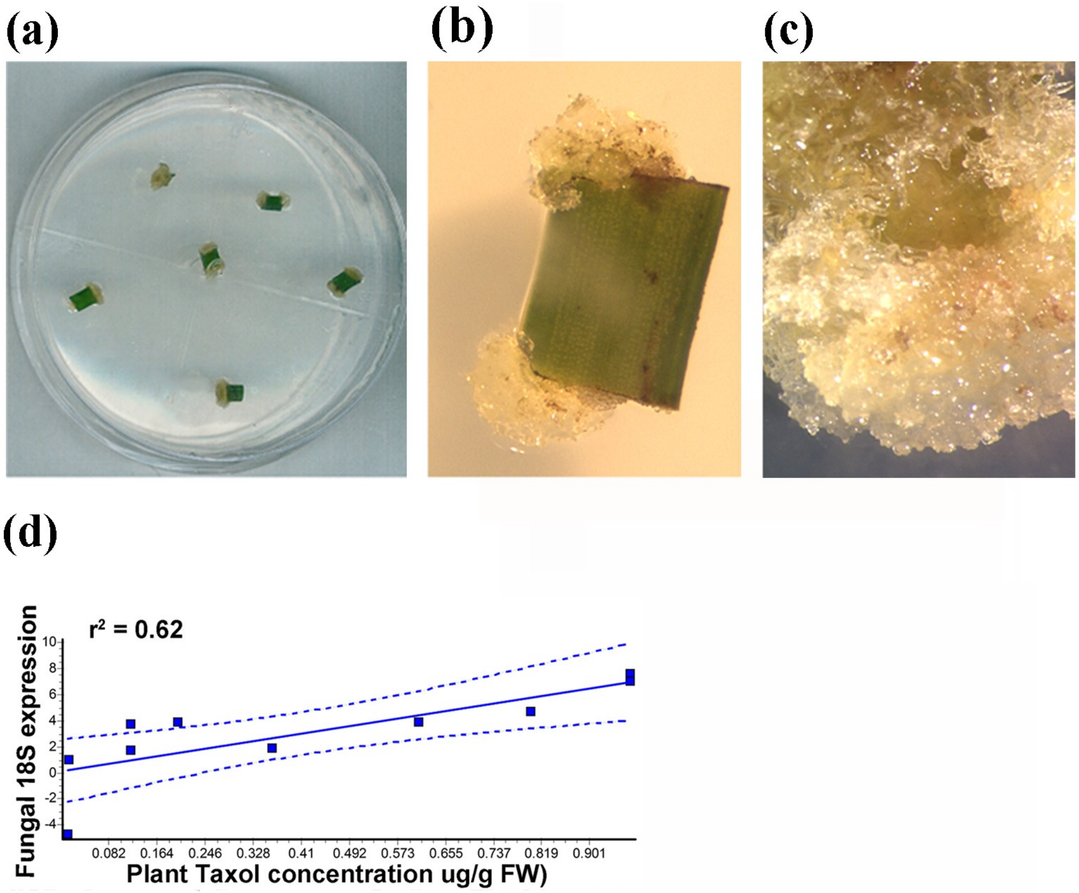
(a)
(b)
(c)
(d)
r² = 0.62
Fungal 18S expression
10
8
6
4
2
0
-2
-4
0.082 0.164 0.246 0.328 0.41 0.492 0.573 0.655 0.737 0.819 0.901
Plant Taxol concentration ug/g FW)
