# Supplementary material for: A fungal endophyte induces transcription of genes encoding a redundant fungicide pathway in its host plant
Source: BMC Plant Biol. 2013 Jun 26;13:93. doi: 10.1186/1471-2229-13-93 (PMC3700885; doi:10.1186/1471-2229-13-93)
Supplement: Supplementary file 5 [file 1471-2229-13-93-S2.doc]

>Paraconiothyrium spp. ITS sequence

TTCCTCCGCTTATTGATATGCTTAAGTTCAGCGGGTATCC
CTACCTGATCCGAGGTCAAAGACGGTAATGTTGCTTCGTG
GACGCGGGCCGCGCACCTCGAGAAGCGCAATGTGCTGCGC
GAGAGGAGGCAAGGACCGCTGCCAATGAATTTGGGGCGAG
TCCGCGCGCAGAGGCGGGACAGACGCCCAACACCAAGCAG
AGCTTGAGGGTGTAGATGACGCTCGAACAGGCATGCCCCA
TGGAATACCAAGGGGCGCAATGTGCGTTCAAAGATTCGAT
GATTCACTGAATTCTGCAATTCACACTACTTATCGCATTT
CGCTGCGTTCTTCATCGATGCCAGAGCCAAGAGATCCATT
GTTGAAAGTTGTAACGATTGTTTGTATCAGAACAGGTAAT
GCTAGATGCAAAAAAAGGTTTTGATAGGTTCCAACGGCAG
GTTGCCCCGCCGAAGGAGAACGAAAGGTGCTCGTAAAAAA
AGGATTCAGACATGCGGCGCGTGAAAGTGTTACCTCTACC
GCCCGACGGCAGCTGTTGCTCCCGCCGAGGGCCGCGACCG
CACCTCATGGAATAGATAATGATCCTTCCGCAGGTTCACC
TACGGA
