# Supplementary material for: A fungal endophyte induces transcription of genes encoding a redundant fungicide pathway in its host plant
Source: BMC Plant Biol. 2013 Jun 26;13:93. doi: 10.1186/1471-2229-13-93 (PMC3700885; doi:10.1186/1471-2229-13-93)
Supplement: Supplementary file 6 [file 1471-2229-13-93-S3.doc]

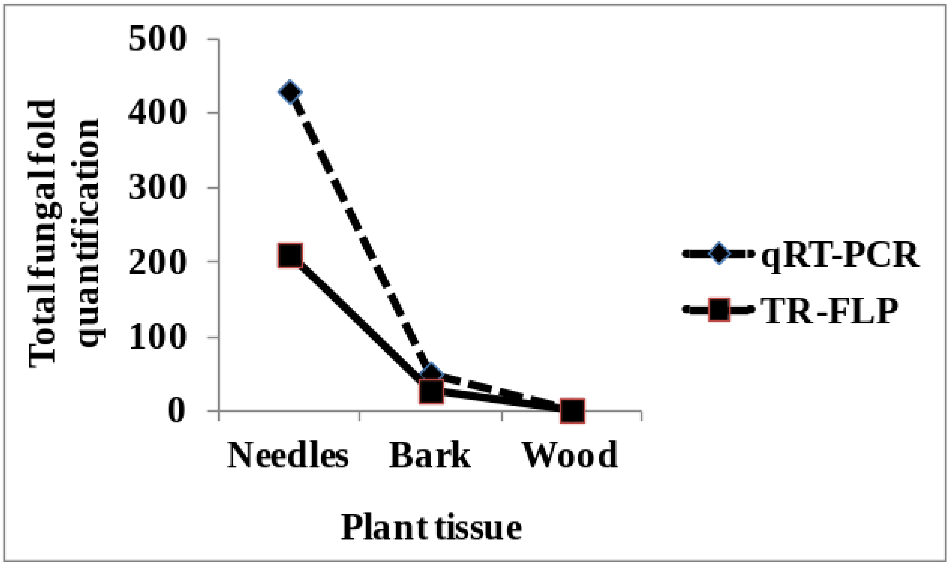

Total fungal fold quantification
500
400
300
200
100
0
Needles
Bark
Wood
Plant tissue
qRT-PCR
TR-FLP
