# Supplementary material for: A fungal endophyte induces transcription of genes encoding a redundant fungicide pathway in its host plant
Source: BMC Plant Biol. 2013 Jun 26;13:93. doi: 10.1186/1471-2229-13-93 (PMC3700885; doi:10.1186/1471-2229-13-93)
Supplement: Supplementary file 7 [file 1471-2229-13-93-S5.doc]

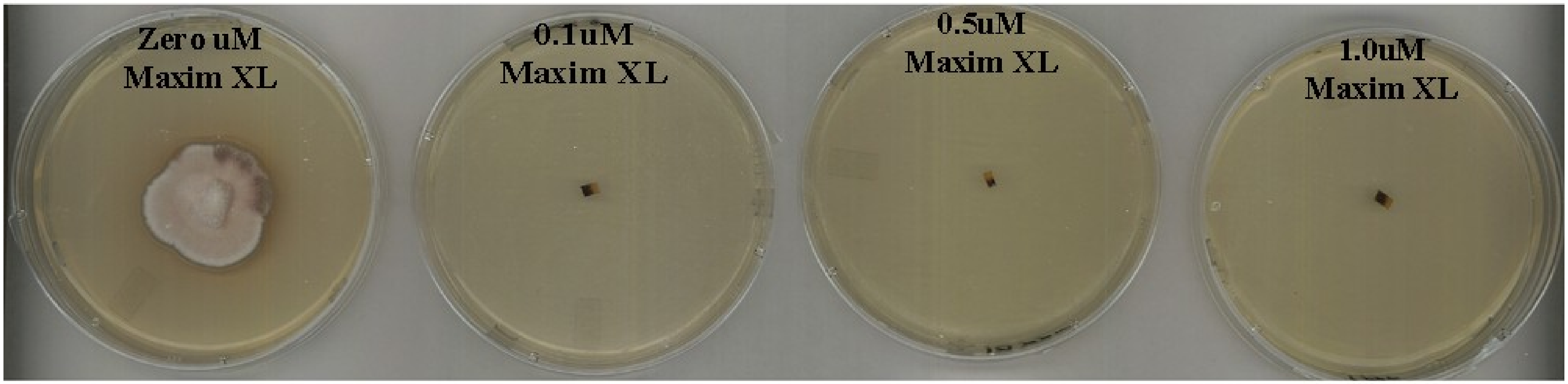
Zero uM
Maxim XL
0.1uM
Maxim XL
0.5uM
Maxim XL
1.0uM
Maxim XL
