# Supplementary material for: A fungal endophyte induces transcription of genes encoding a redundant fungicide pathway in its host plant
Source: BMC Plant Biol. 2013 Jun 26;13:93. doi: 10.1186/1471-2229-13-93 (PMC3700885; doi:10.1186/1471-2229-13-93)
Supplement: Supplementary file 9 [file 1471-2229-13-93-S7.doc]

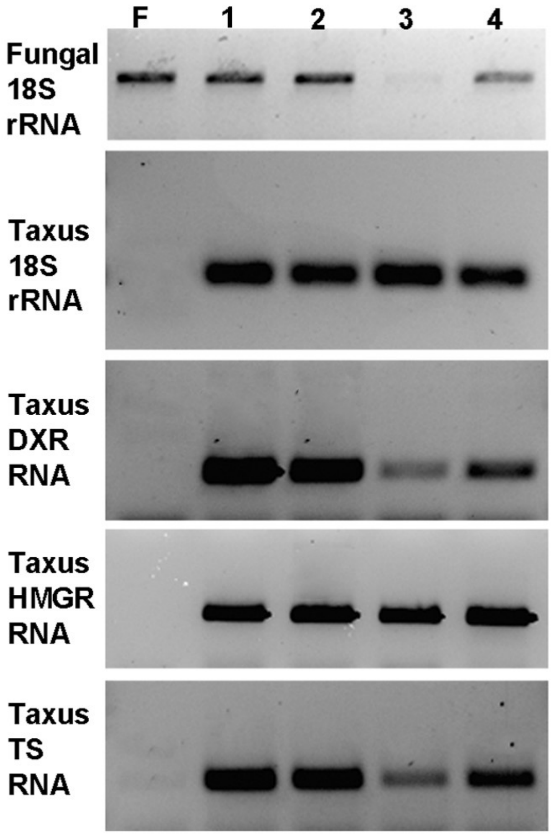
F
1
2
3
4
Fungal 18S rRNA
Taxus 18S rRNA
Taxus DXR RNA
Taxus HMGR RNA
Taxus TS RNA
